# Supplementary material for: Cu(II)-Catalyzed C-N Coupling of (Hetero)aryl Halides and N-Nucleophiles Promoted by α-Benzoin Oxime
Source: Molecules. 2019 Nov 18;24(22):4177. doi: 10.3390/molecules24224177 (PMC6891672; doi:10.3390/molecules24224177)
Supplement: Supplementary file 1 [file molecules-24-04177-s001.pdf]

# Cu (II)-Catalyzed C-N Coupling of (Hetero)aryl Halides and *N*-Nucleophiles Promoted by α-Benzoin Oxime

Chunling Yuan *, Lei Zhang and Yingdai Zhao

Department of Medicinal Chemistry, Pharmacy School, Jinzhou Medical University, Jinzhou 121001, Liaoning, PR China
*Correspondence: yuanchunling@jzmu.edu.cn; Tel.: +86-0416-4673440, +86-13898354237

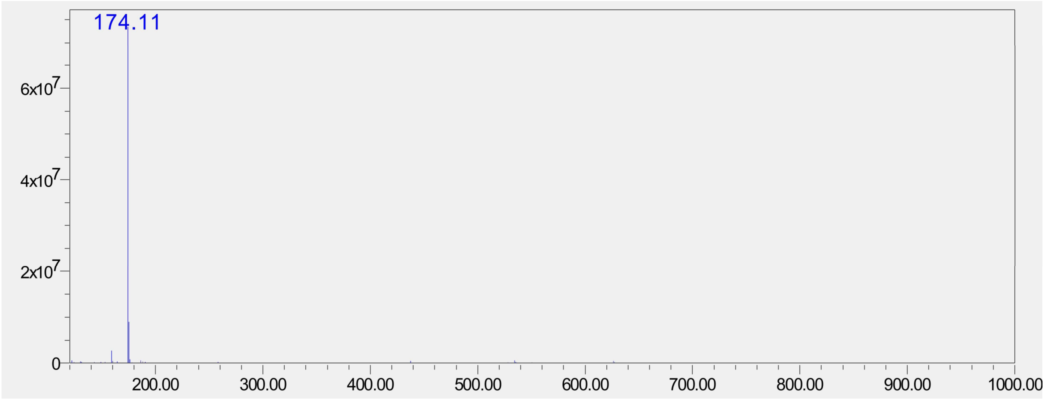
174.11
6x10^7
4x10^7
2x10^7
0
200.00
300.00
400.00
500.00
600.00
700.00
800.00
900.00
1000.00

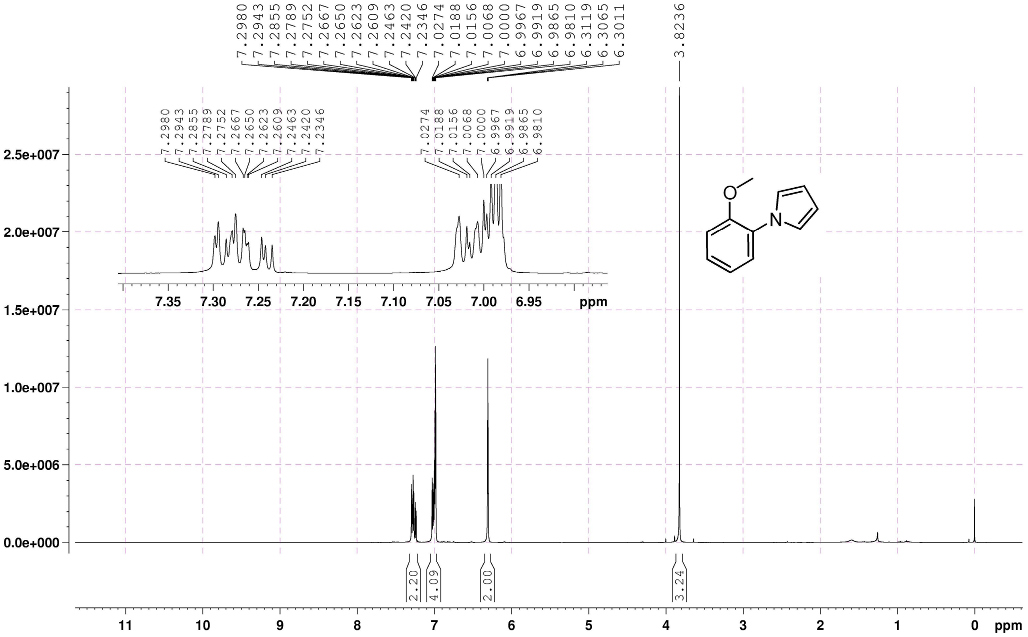
7.2980
7.2943
7.2855
7.2789
7.2752
7.2667
7.2650
7.2623
7.2609
7.2463
7.2420
7.2346
7.0274
7.0188
7.0156
7.0068
7.0000
6.9967
6.9919
6.9865
6.9810
6.3119
6.3065
6.3011
3.8236
2.20
4.09
2.00
3.24
ppm

3a

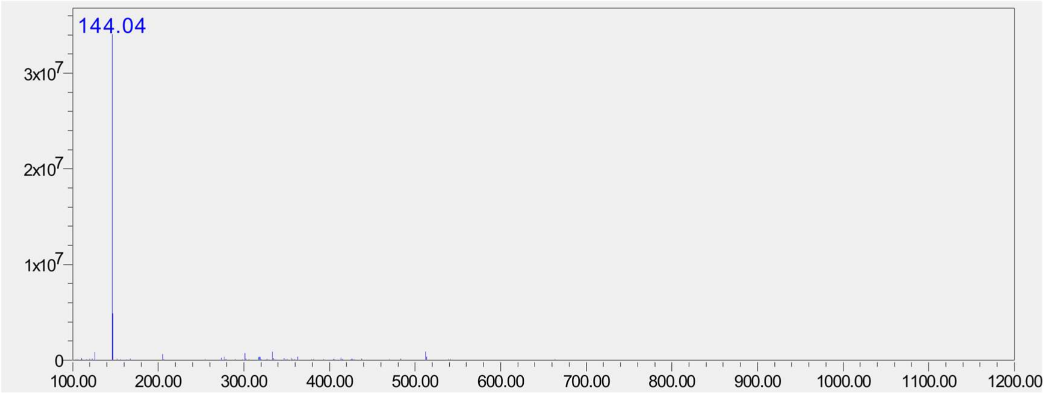
144.04
3x10^7
2x10^7
1x10^7
0
100.00
200.00
300.00
400.00
500.00
600.00
700.00
800.00
900.00
1000.00
1100.00
1200.00

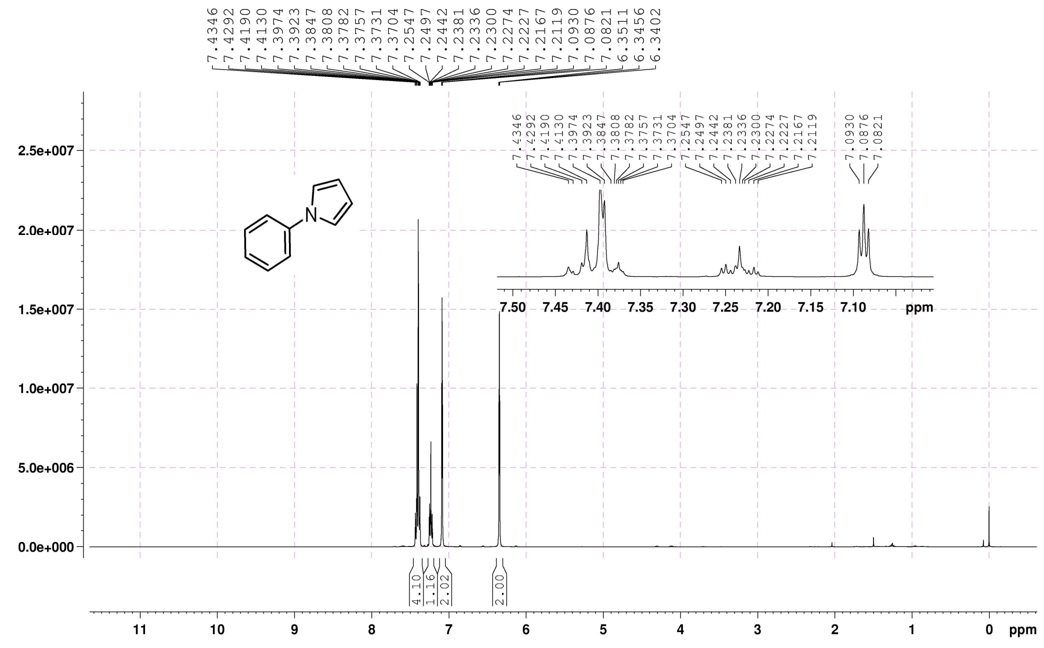
7.4346
7.4292
7.4190
7.4130
7.3974
7.3923
7.3847
7.3808
7.3782
7.3757
7.3731
7.3704
7.2547
7.2497
7.2442
7.2381
7.2336
7.2300
7.2274
7.2227
7.2167
7.2119
7.0930
7.0876
7.0821
6.3511
6.3456
6.3402
N
4.10
1.16
2.02
2.00
ppm

**3b**

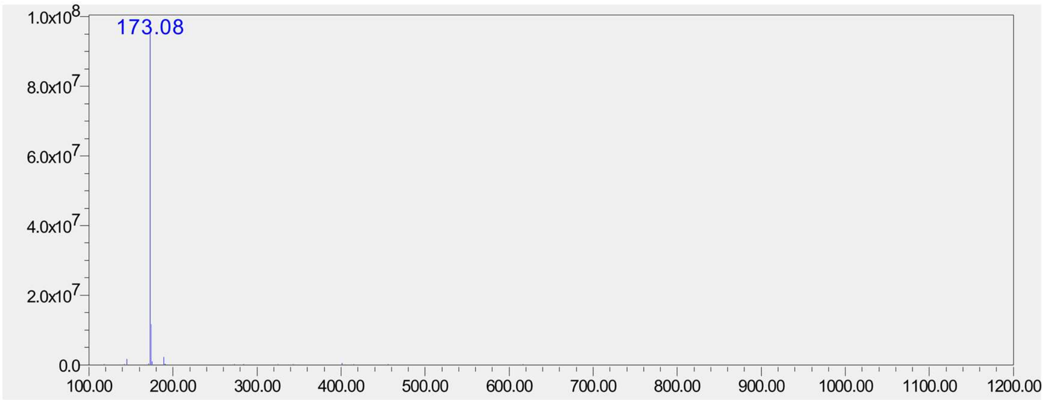
173.08

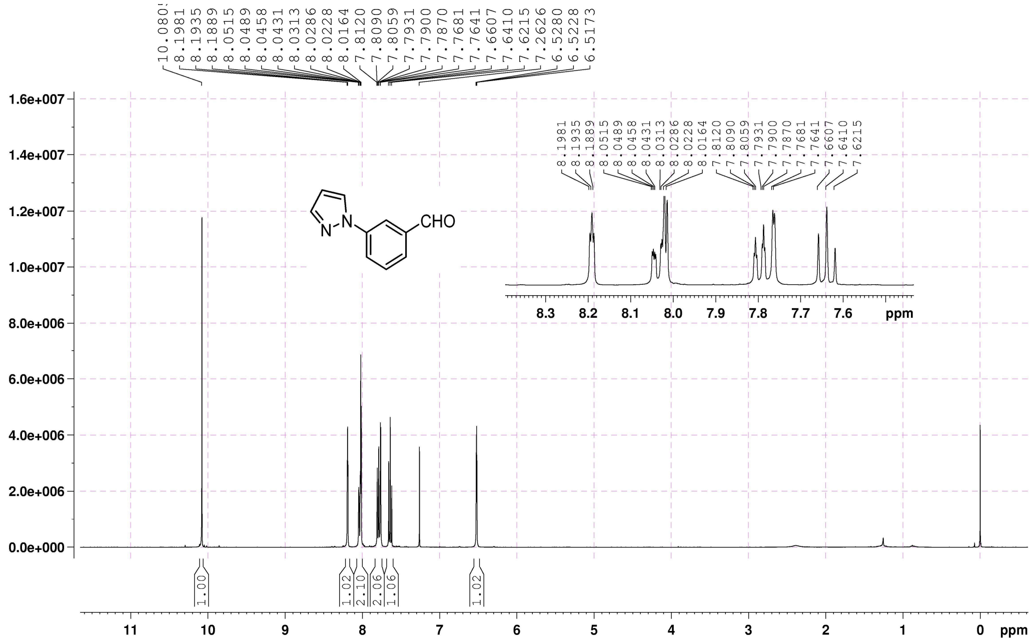

**3c**

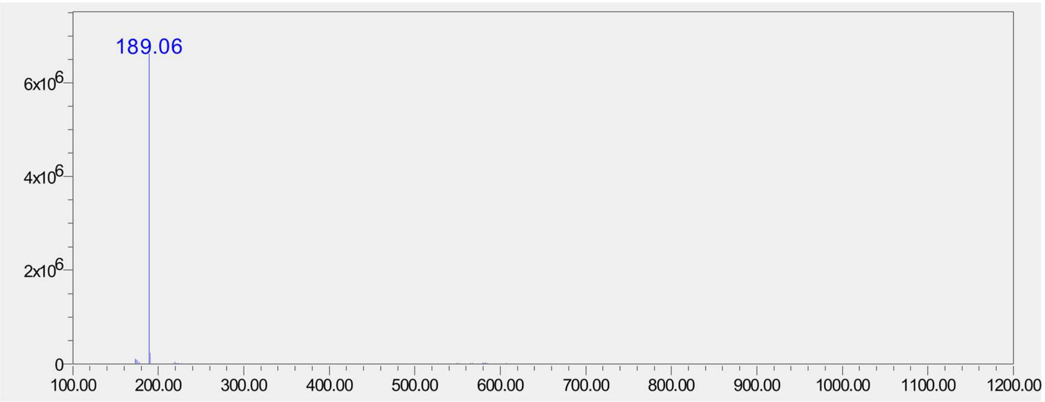

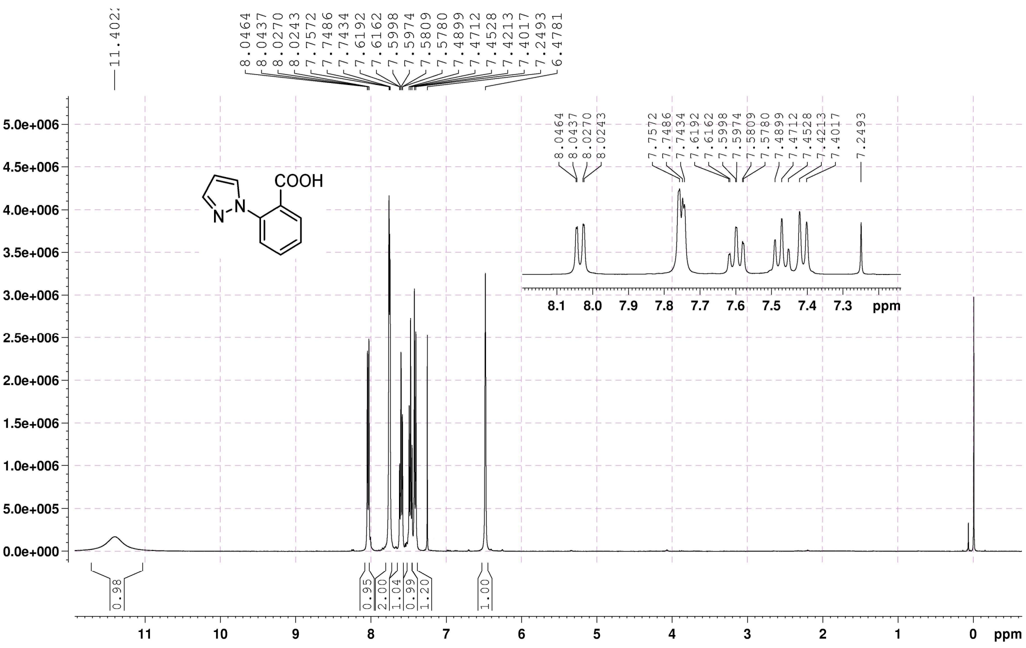

11.4022
8.0464
8.0437
8.0270
8.0243
7.7572
7.7486
7.7434
7.6192
7.6162
7.5998
7.5974
7.5809
7.5780
7.4899
7.4712
7.4528
7.4213
7.4017
7.2493
6.4781
COOH
N
N
ppm
0.98
0.95
2.00
1.04
0.99
1.20
1.00


**3d**

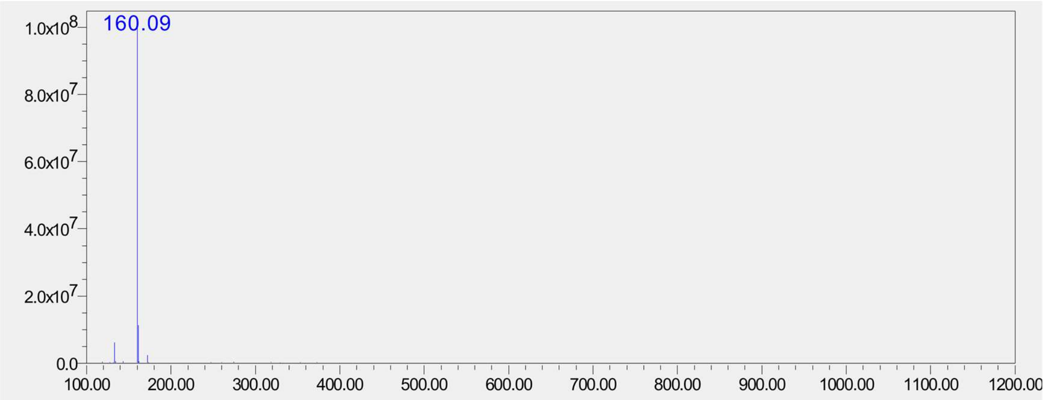

160.09

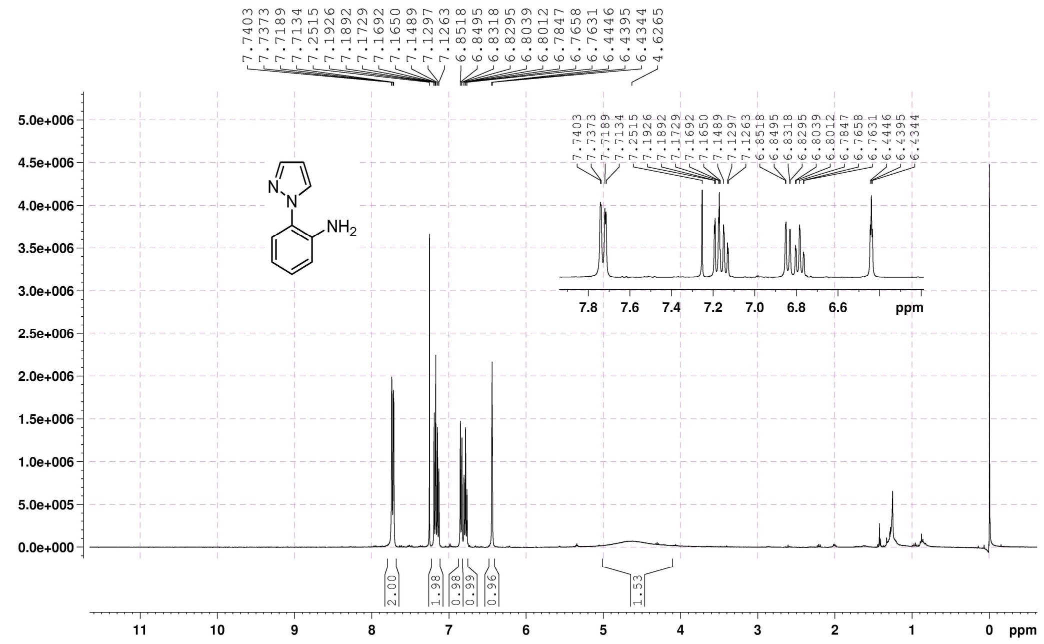
7.7403
7.7373
7.7189
7.7134
7.2515
7.1926
7.1892
7.1729
7.1692
7.1650
7.1489
7.1297
7.1263
6.8518
6.8495
6.8318
6.8295
6.8039
6.8012
6.7847
6.7658
6.7631
6.4446
6.4395
6.4344
4.6265
NH2
5.0e+006
4.5e+006
4.0e+006
3.5e+006
3.0e+006
2.5e+006
2.0e+006
1.5e+006
1.0e+006
5.0e+005
0.0e+000
2.00
1.98
0.98
0.99
0.96
1.53
11
10
9
8
7
6
5
4
3
2
1
0
ppm
7.8
7.6
7.4
7.2
7.0
6.8
6.6
ppm

**3e**

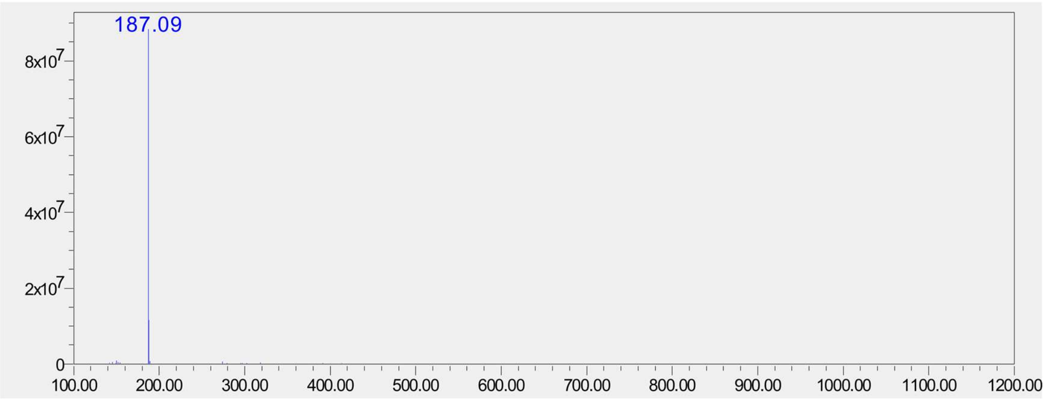
187.09
8x10^7
6x10^7
4x10^7
2x10^7
0
100.00
200.00
300.00
400.00
500.00
600.00
700.00
800.00
900.00
1000.00
1100.00
1200.00

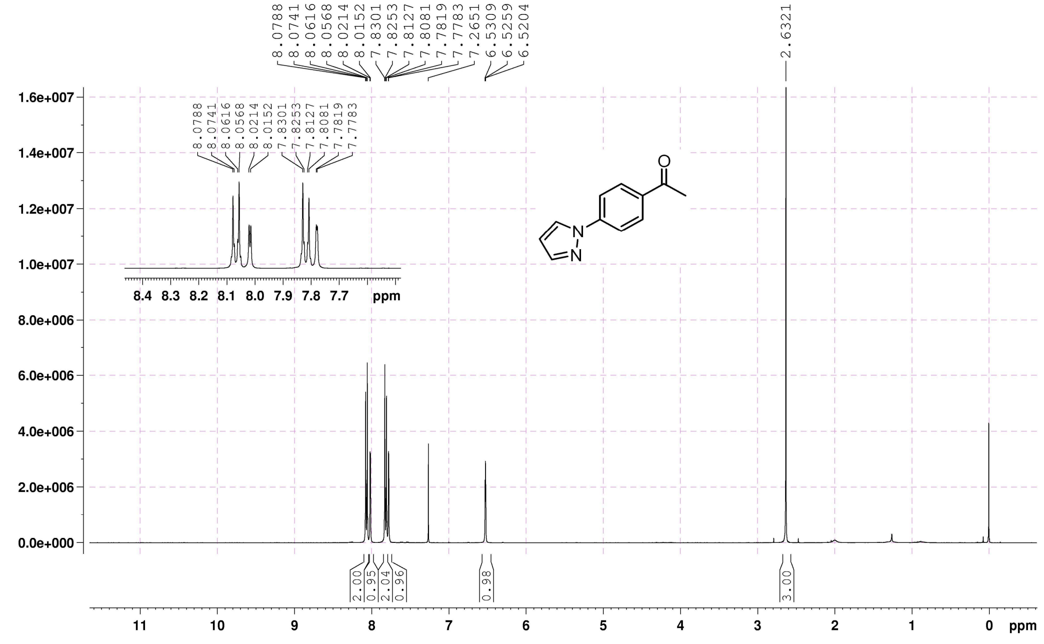
8.0788
8.0741
8.0616
8.0568
8.0214
8.0152
7.8301
7.8253
7.8127
7.8081
7.7819
7.7783
7.2651
6.5309
6.5259
6.5204
2.6321
2.00
0.95
2.04
0.96
0.98
3.00
ppm

**3f**

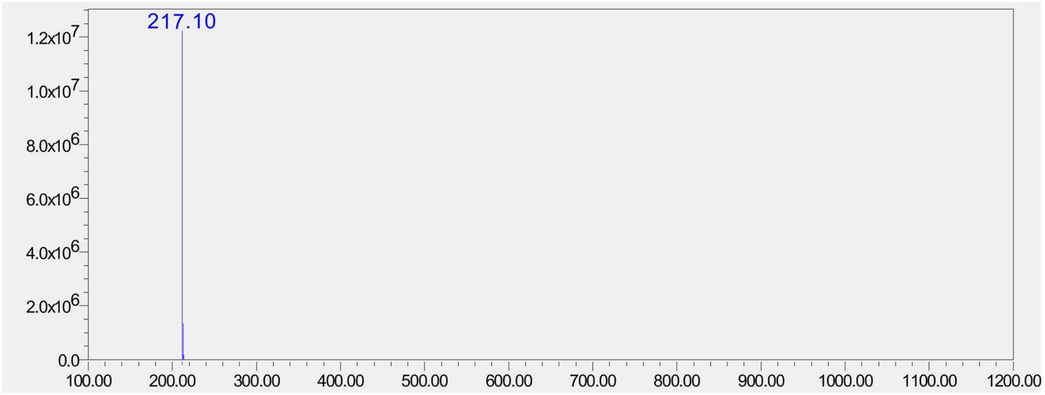
217.10

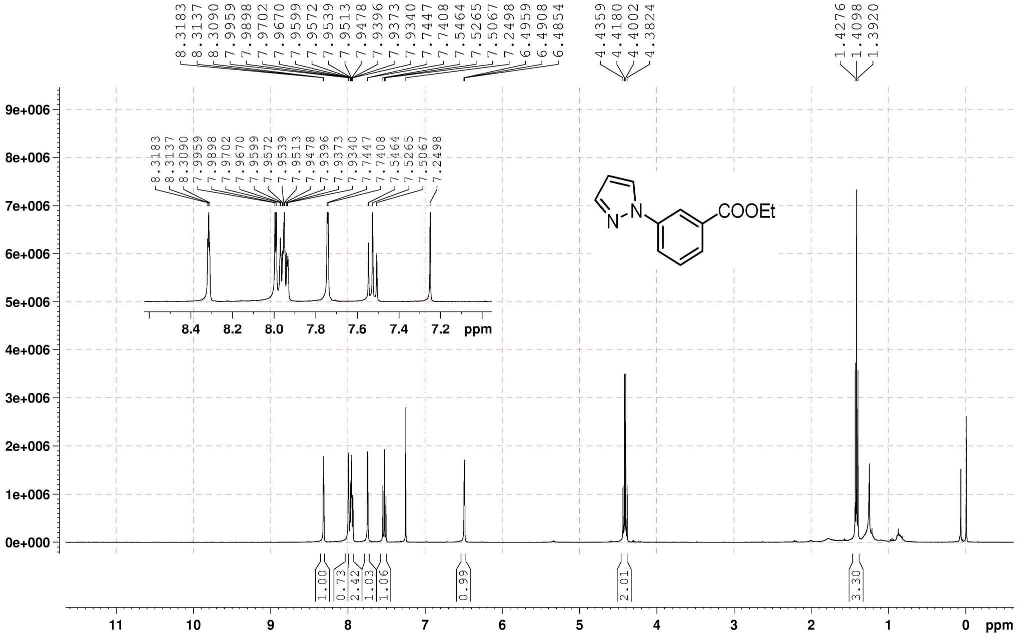


**3g**

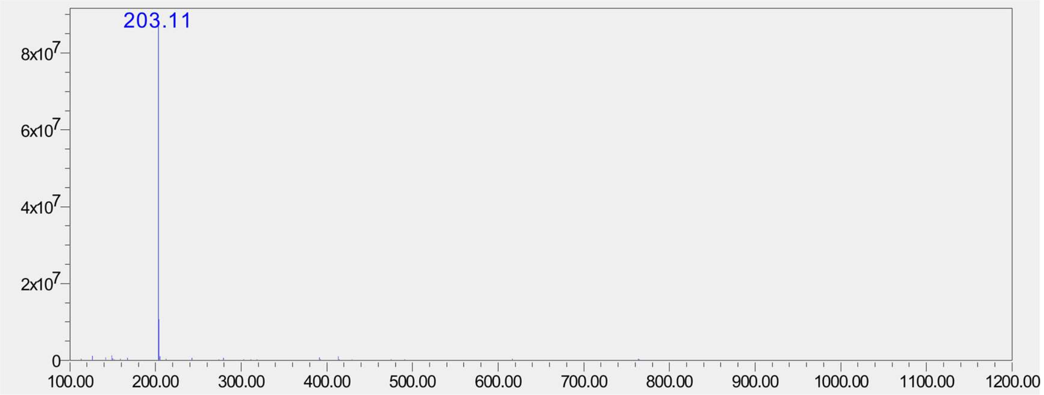

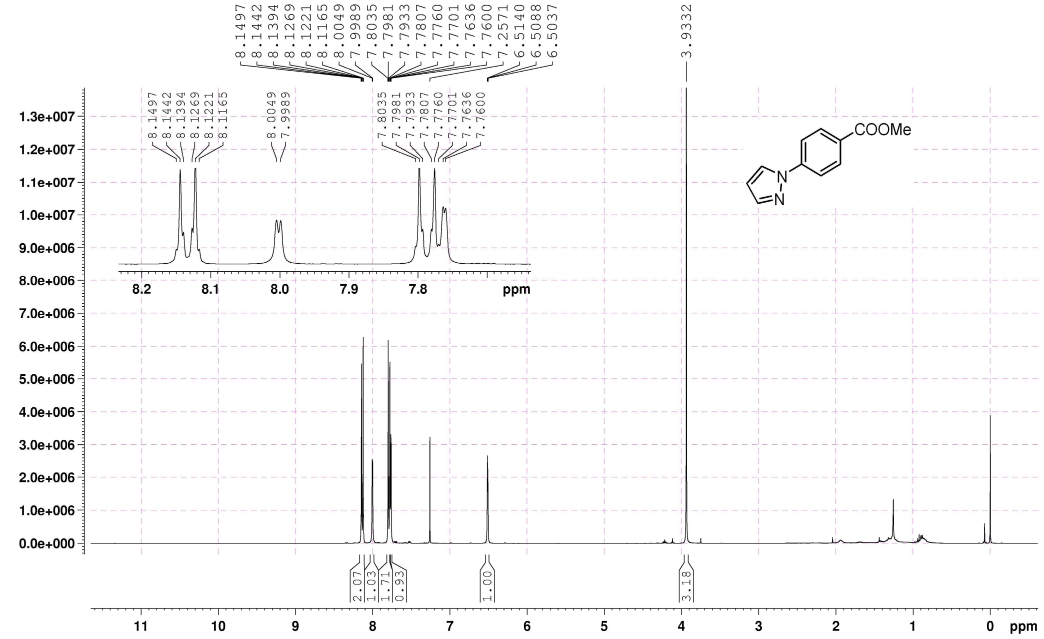

8.1497
8.1442
8.1394
8.1269
8.1221
8.1165
8.0049
7.9989
7.8035
7.7981
7.7933
7.7807
7.7760
7.7701
7.7636
7.7600
7.2571
6.5140
6.5088
6.5037
3.9332
COOMe
N
N
2.07
1.03
1.71
0.93
1.00
3.18
ppm


**3h**

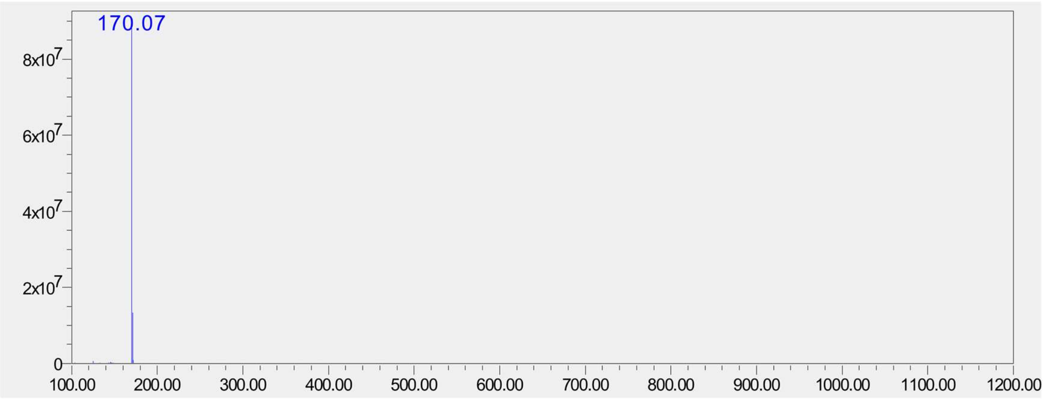

170.07

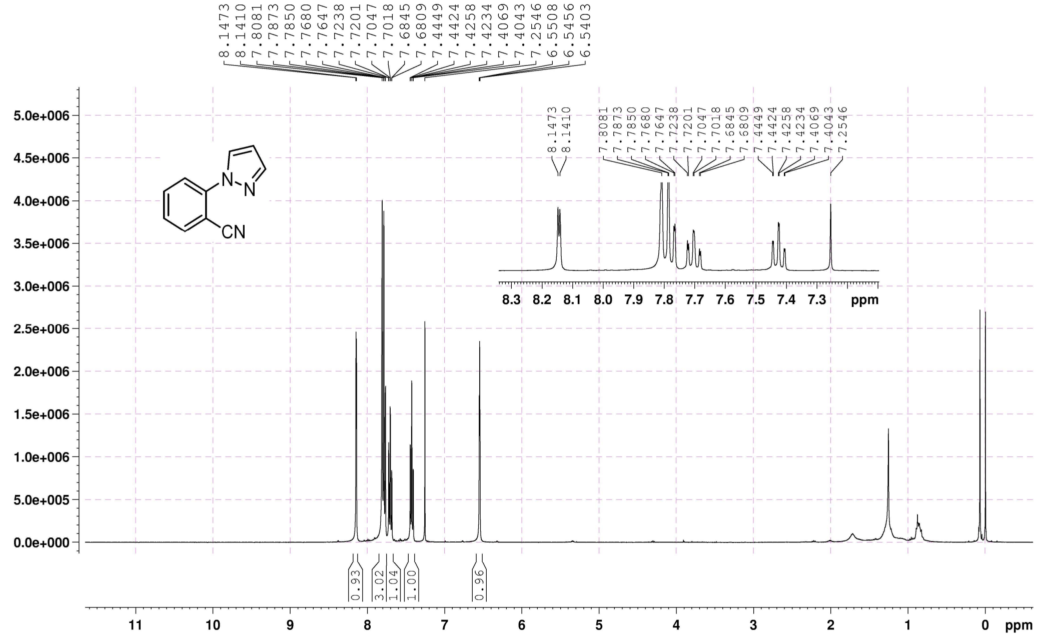

8.1473
8.1410
7.8081
7.7873
7.7850
7.7680
7.7647
7.7238
7.7201
7.7047
7.7018
7.6845
7.6809
7.4449
7.4424
7.4258
7.4234
7.4069
7.4043
7.2546
6.5508
6.5456
6.5403
CN
N
N
0.93
3.02
1.04
1.00
0.96
ppm


**3i**

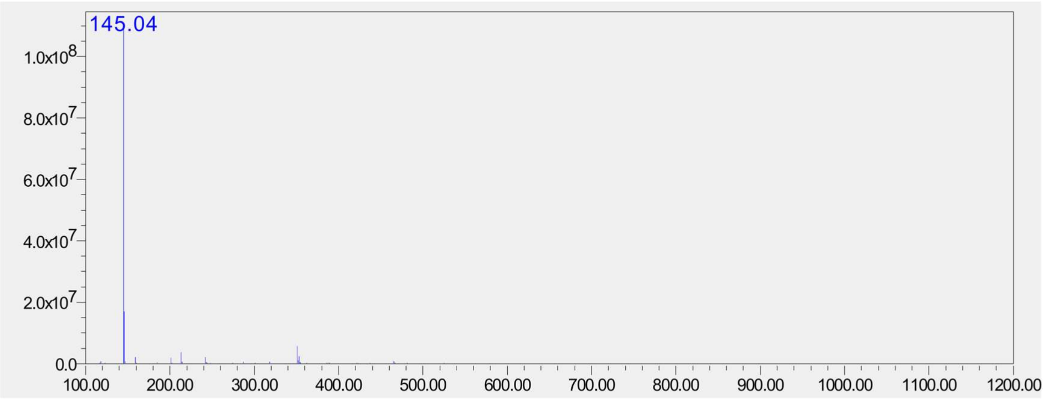

145.04

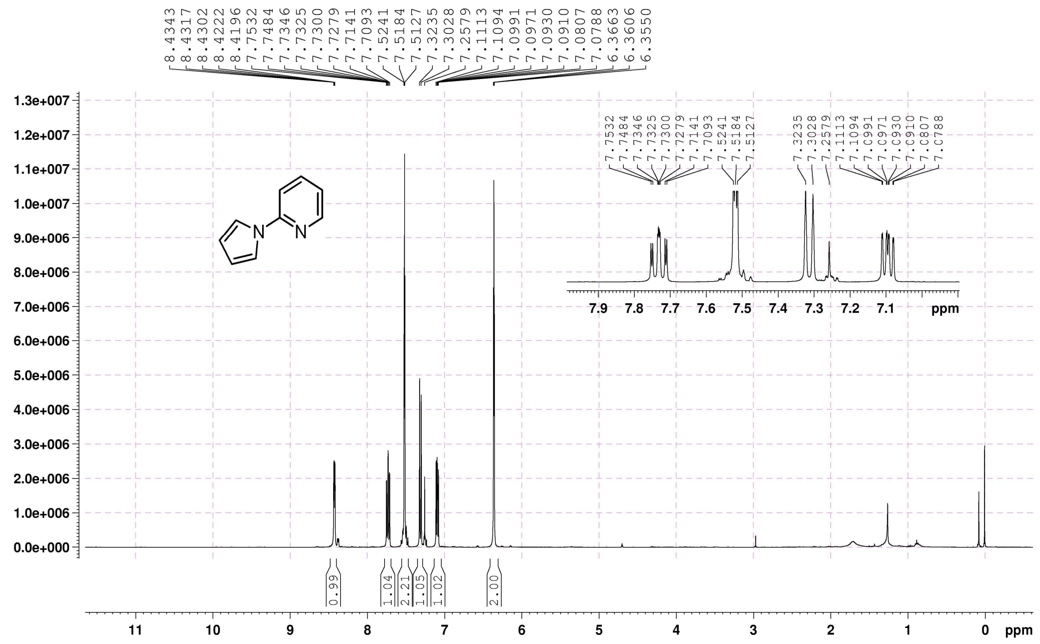

**3j**

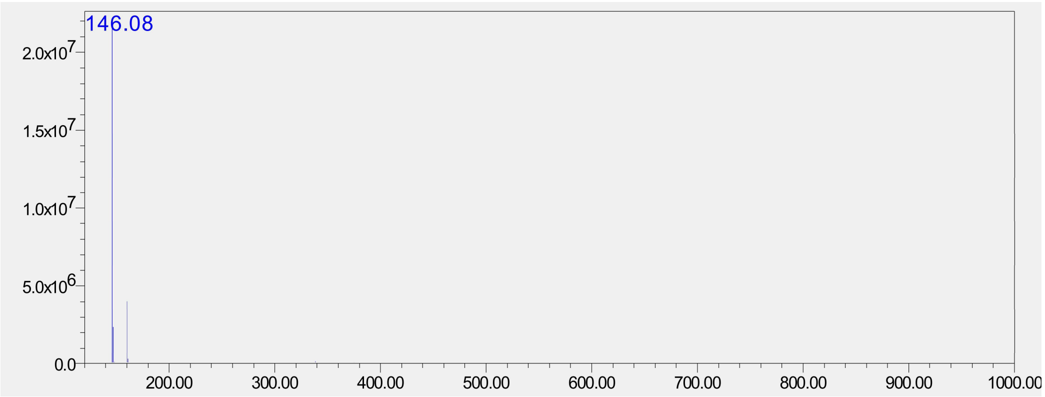

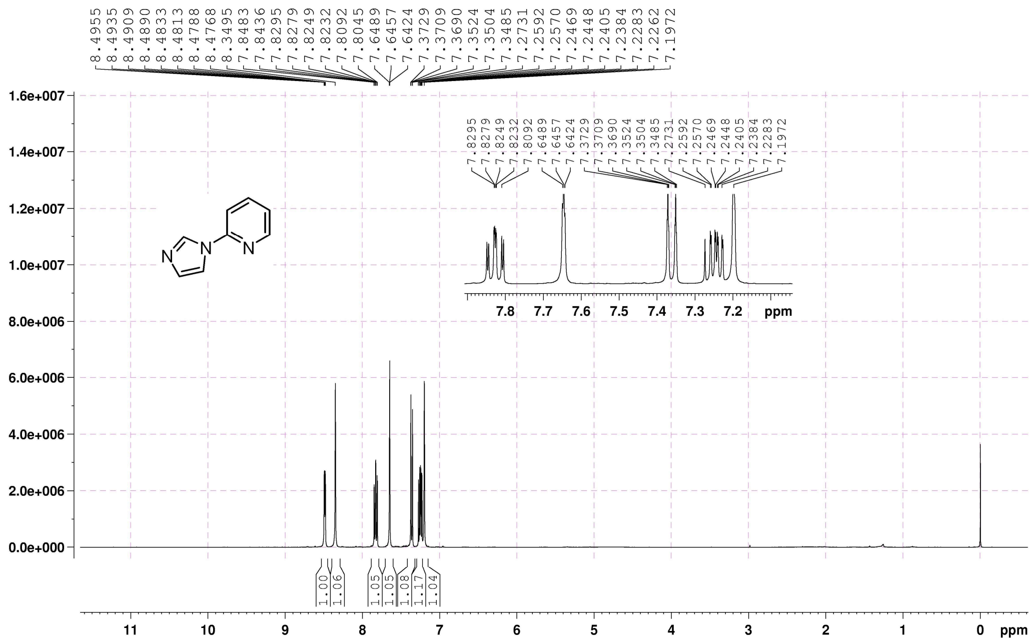
8.4955
8.4935
8.4909
8.4890
8.4833
8.4813
8.4788
8.4768
8.3495
8.3483
7.8436
7.8295
7.8279
7.8249
7.8232
7.8092
7.8045
7.6489
7.6457
7.6424
7.3729
7.3709
7.3690
7.3524
7.3504
7.3485
7.2731
7.2592
7.2570
7.2469
7.2448
7.2405
7.2384
7.2283
7.2262
7.1972
N
N
N
1.00
1.06
1.05
1.05
1.08
1.17
1.04
ppm

**3k**

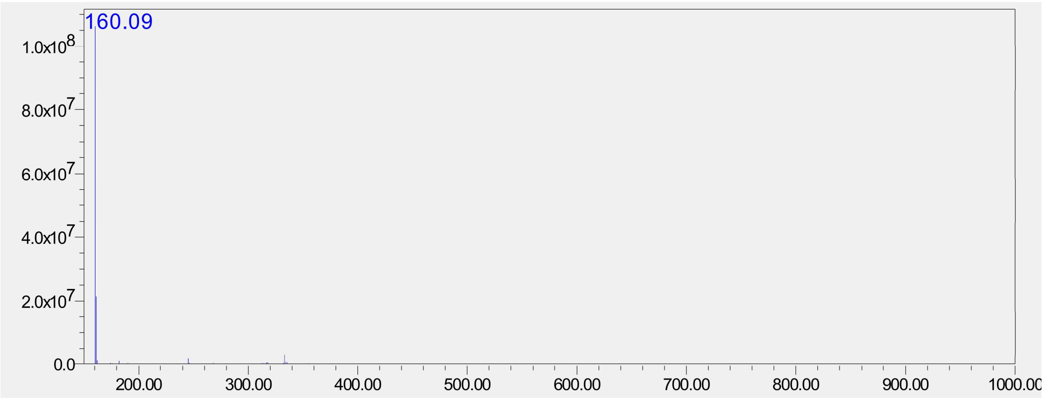
160.09

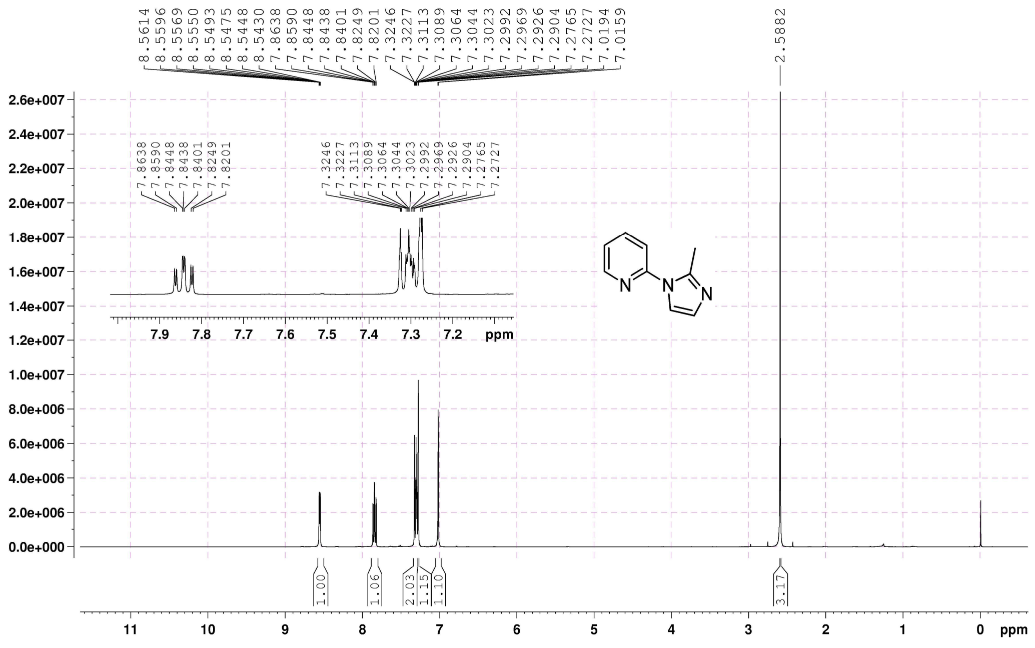
8.5614
8.5596
8.5569
8.5550
8.5493
8.5475
8.5448
8.5430
7.8638
7.8590
7.8448
7.8438
7.8401
7.8249
7.8201
7.3246
7.3227
7.3113
7.3089
7.3064
7.3044
7.3023
7.2992
7.2969
7.2926
7.2904
7.2765
7.2727
7.0194
7.0159
2.5882
N
N
N
1.00
1.06
2.03
1.15
1.10
3.17
ppm

**3l**

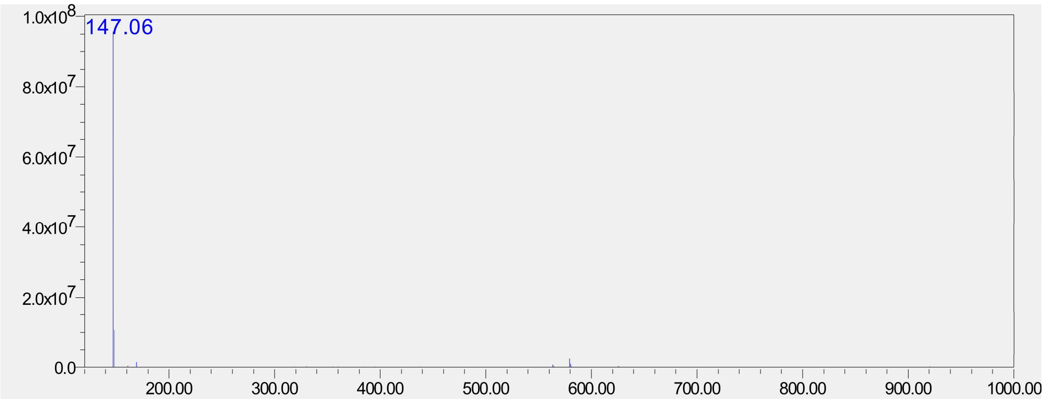
147.06

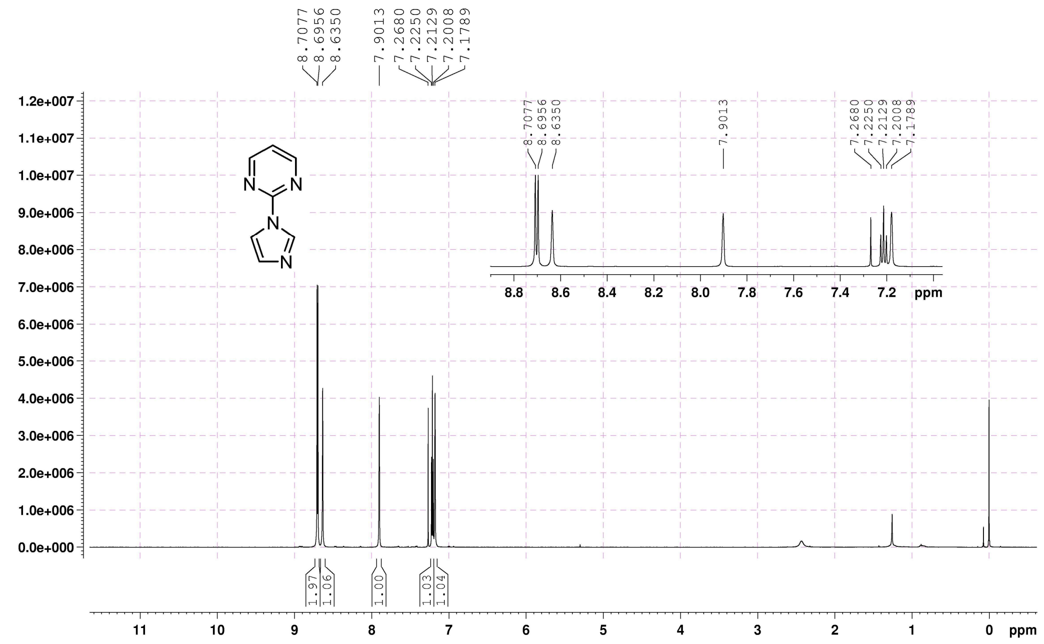
8.7077
8.6956
8.6350
7.9013
7.2680
7.2250
7.2129
7.2008
7.1789
1.2e+007
1.1e+007
1.0e+007
9.0e+006
8.0e+006
7.0e+006
6.0e+006
5.0e+006
4.0e+006
3.0e+006
2.0e+006
1.0e+006
0.0e+000
N
N
N
N
8.8 8.6 8.4 8.2 8.0 7.8 7.6 7.4 7.2 ppm
1.97
1.06
1.00
1.03
1.04
11 10 9 8 7 6 5 4 3 2 1 0 ppm

**3m**

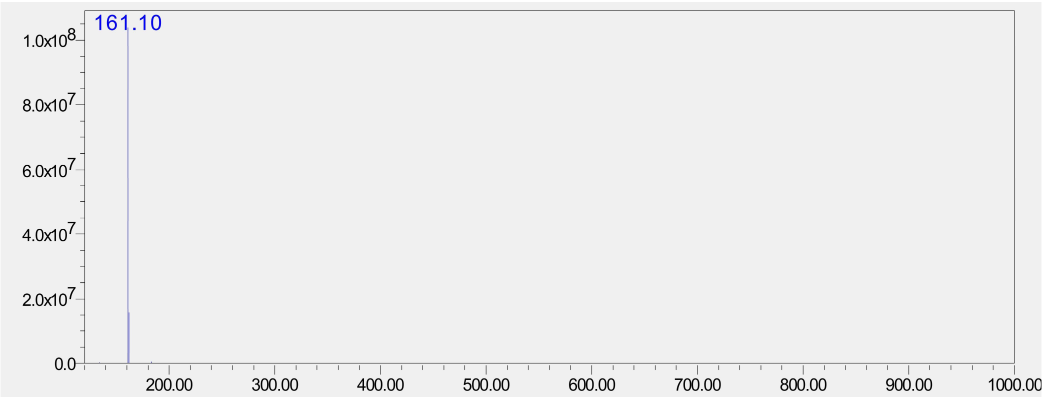
161.10
1.0x10^8
8.0x10^7
6.0x10^7
4.0x10^7
2.0x10^7
0.0
200.00 300.00 400.00 500.00 600.00 700.00 800.00 900.00 1000.00

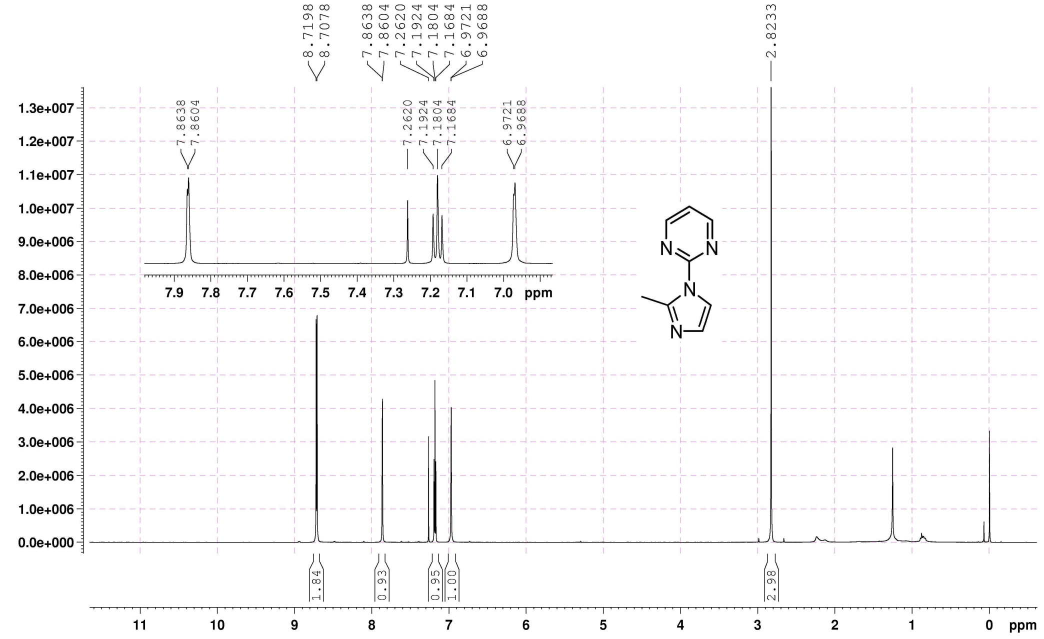

8.7198
8.7078
7.8638
7.8604
7.2620
7.1924
7.1804
7.1684
6.9721
6.9688
2.8233
1.84
0.93
0.95
1.00
2.98
ppm


**3n**

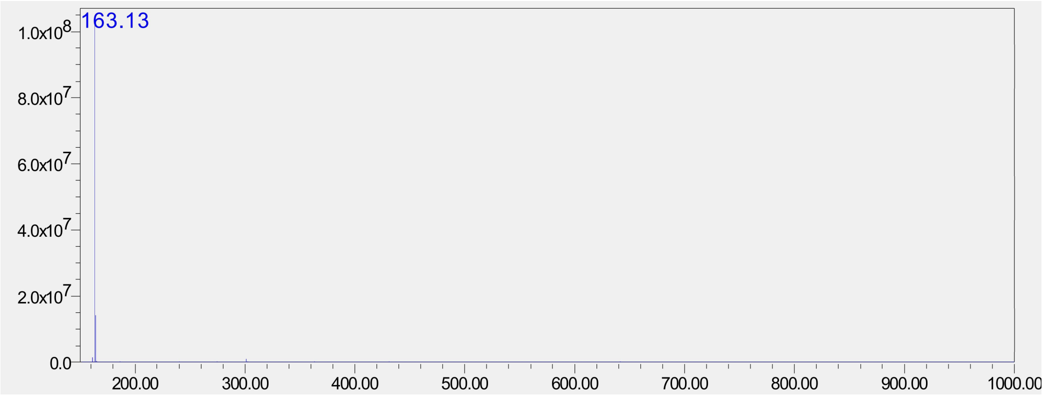

163.13

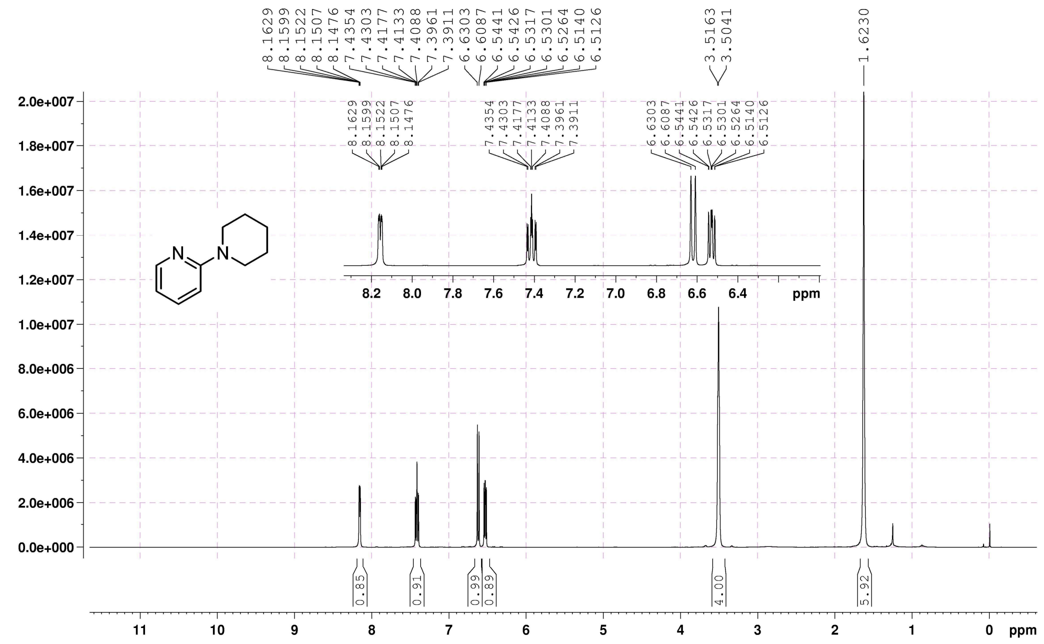
8.1629
8.1599
8.1522
8.1507
8.1476
7.4354
7.4303
7.4177
7.4133
7.4088
7.3961
7.3911
6.6303
6.6087
6.5441
6.5426
6.5317
6.5301
6.5264
6.5140
6.5126
3.5163
3.5041
1.6230
0.85
0.91
0.99
0.89
4.00
5.92
ppm

**4a**

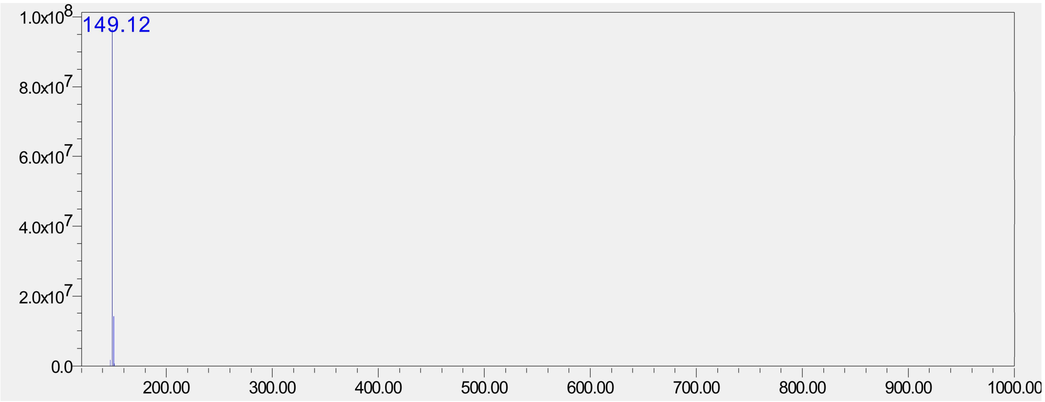
149.12

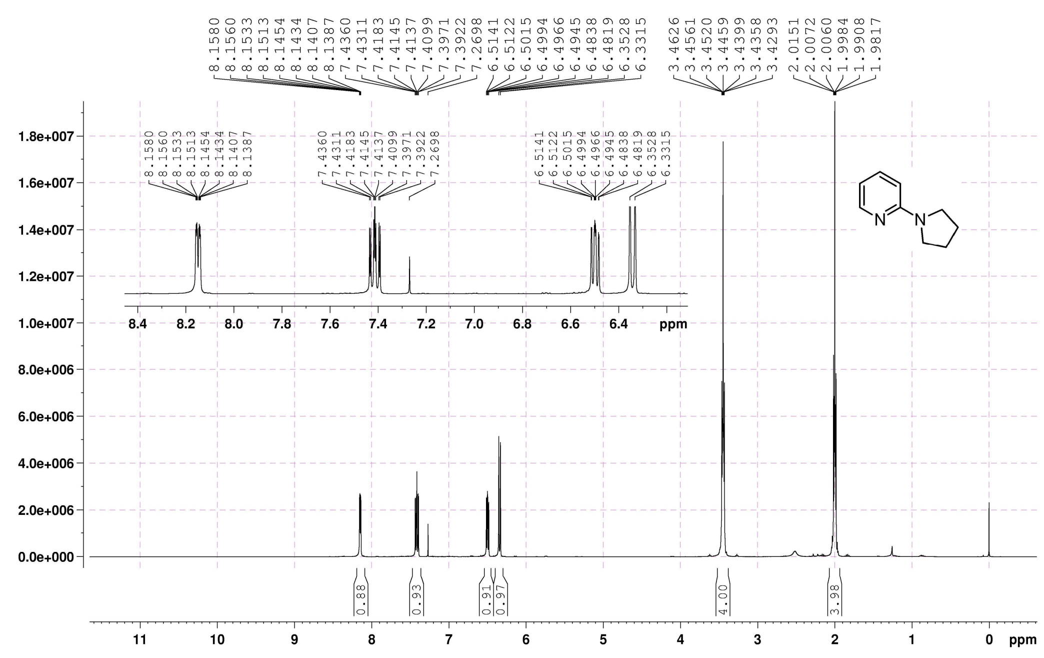

**4b**

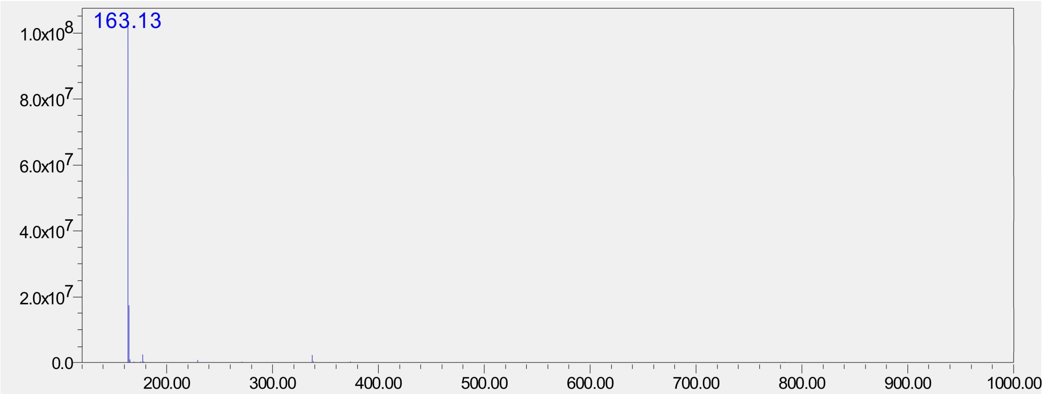

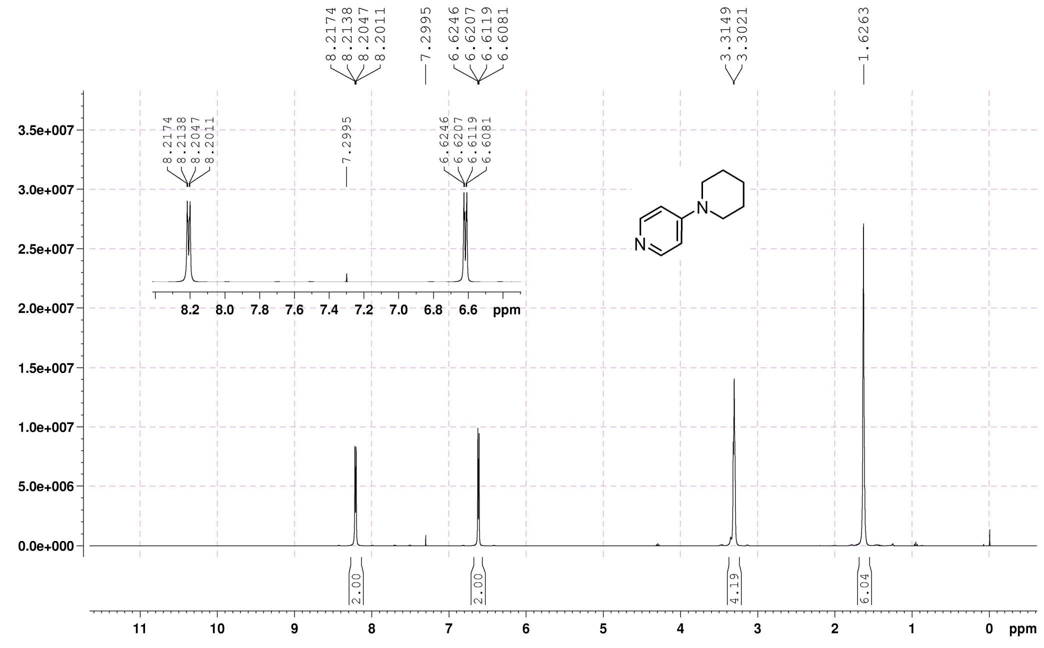

**4c**

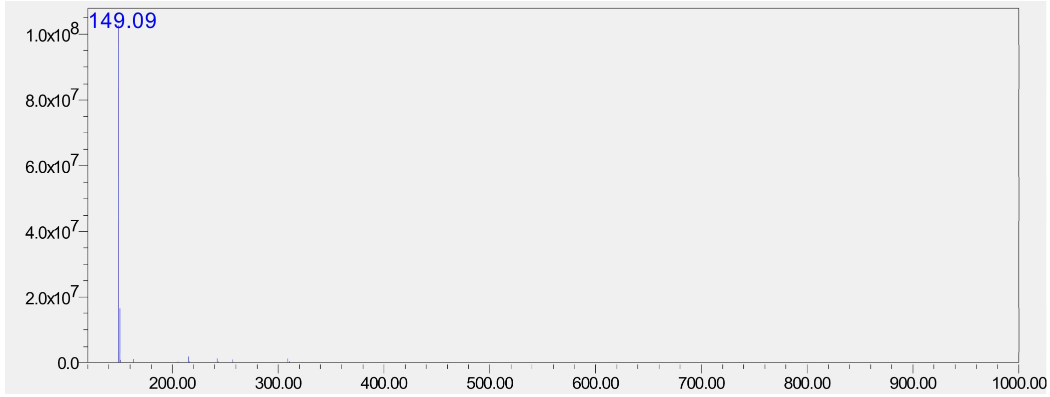

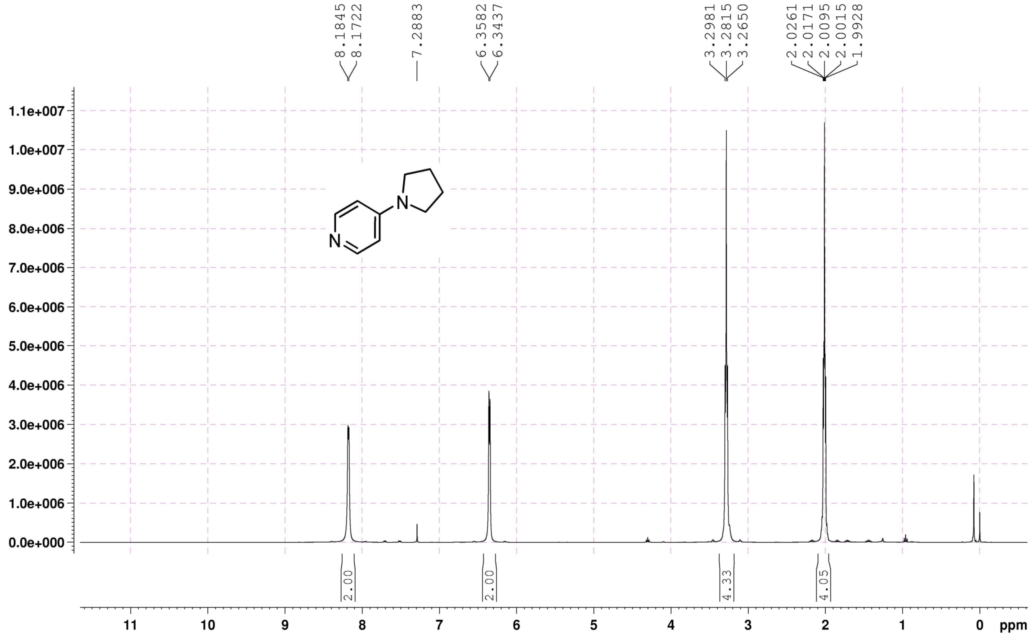

8.1845
8.1722
7.2883
6.3582
6.3437
3.2981
3.2815
3.2650
2.0261
2.0171
2.0095
2.0015
1.9928
N
N
2.00
2.00
4.33
4.05
ppm


**4d**

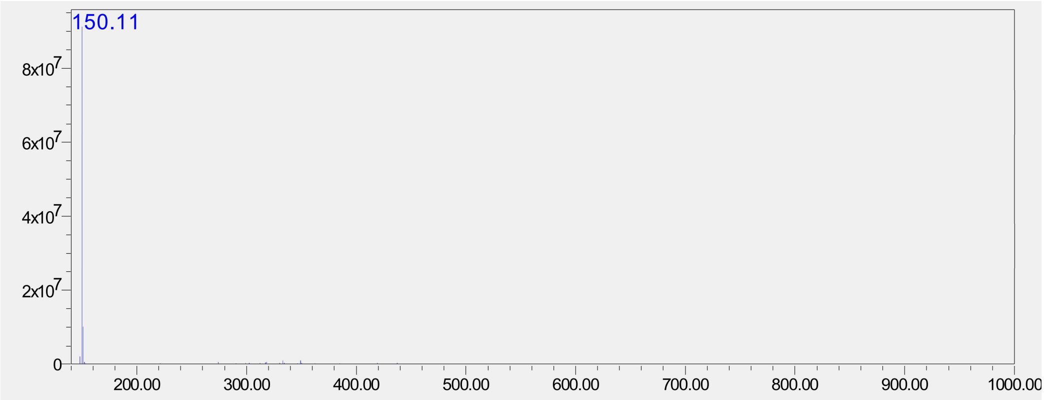

150.11

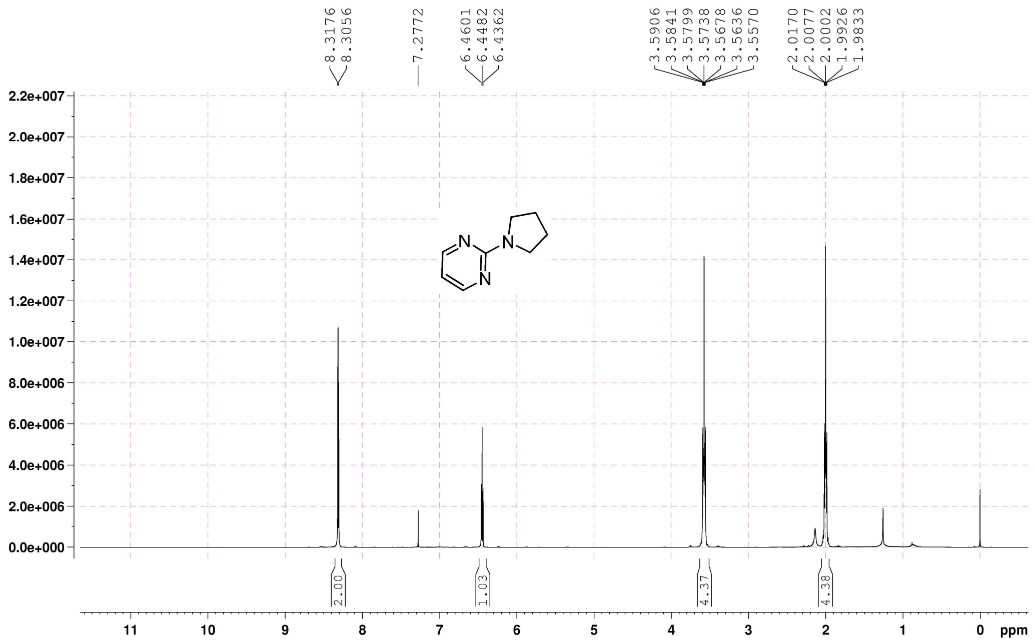
8.3176
8.3056
7.2772
6.4601
6.4482
6.4362
3.5906
3.5841
3.5799
3.5738
3.5678
3.5636
3.5570
2.0170
2.0077
2.0002
1.9926
1.9833
2.2e+007
2.0e+007
1.8e+007
1.6e+007
1.4e+007
1.2e+007
1.0e+007
8.0e+006
6.0e+006
4.0e+006
2.0e+006
0.0e+000
N
N
N
2.00
1.03
4.37
4.38
11 10 9 8 7 6 5 4 3 2 1 0 ppm

**4e**

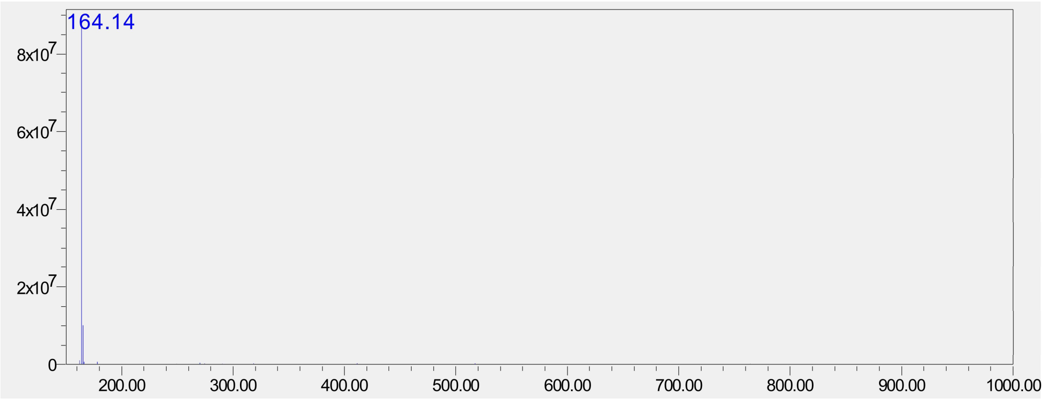
164.14
8x10^7
6x10^7
4x10^7
2x10^7
0
200.00 300.00 400.00 500.00 600.00 700.00 800.00 900.00 1000.00

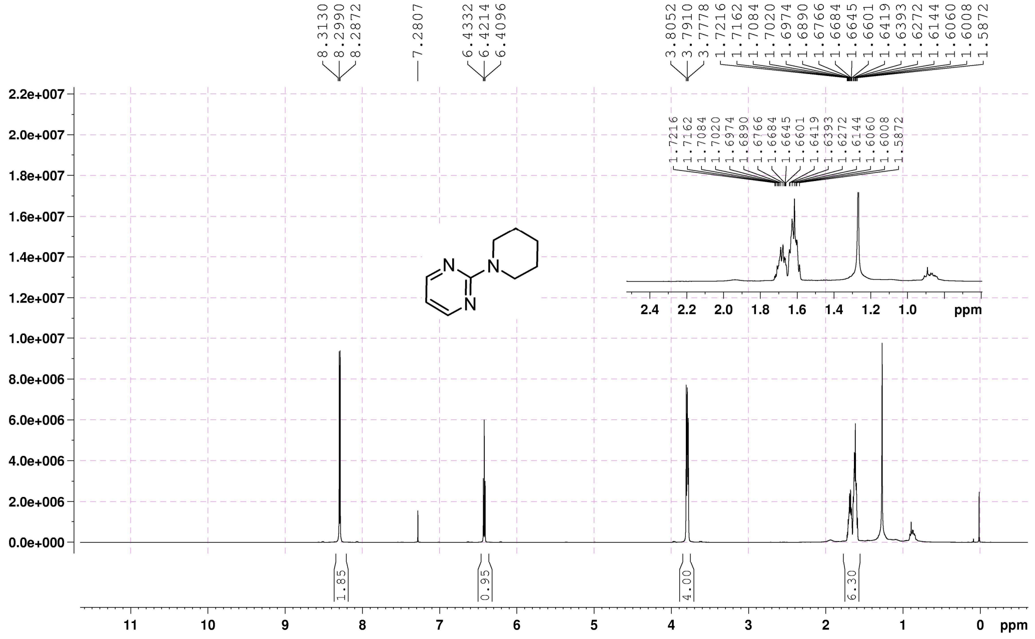
8.3130
8.2990
8.2872
7.2807
6.4332
6.4214
6.4096
3.8052
3.7910
3.7778
1.7216
1.7162
1.7084
1.7020
1.6974
1.6890
1.6766
1.6684
1.6645
1.6601
1.6419
1.6393
1.6272
1.6144
1.6060
1.6008
1.5872
1.85
0.95
4.00
6.30
ppm

**4f**

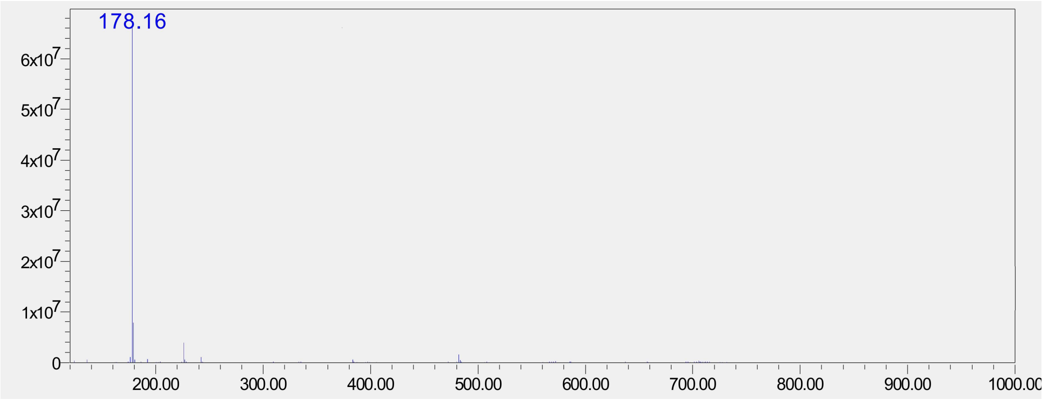
178.16

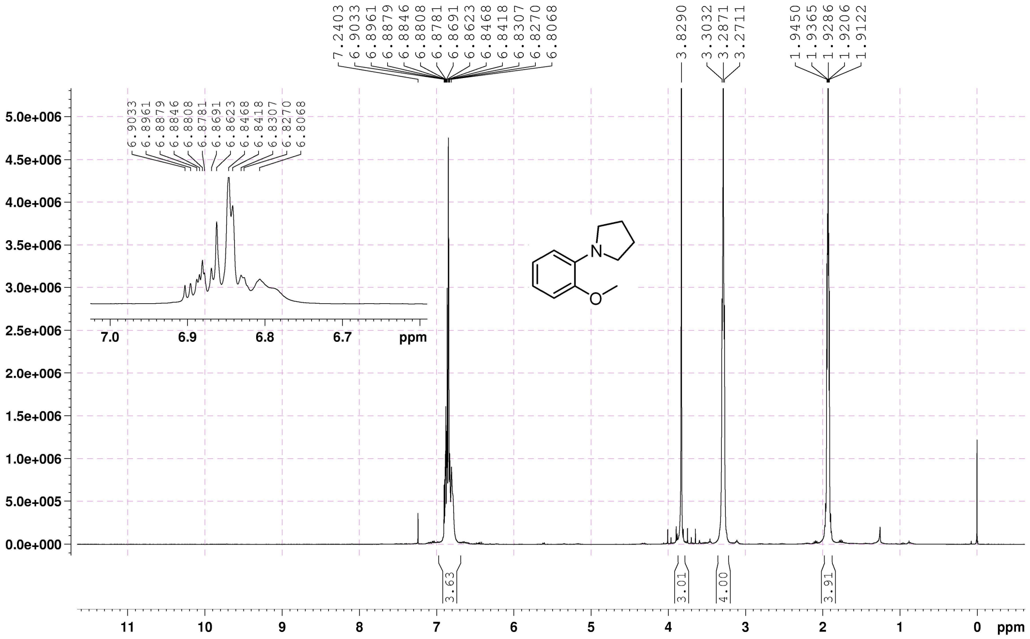
7.2403
6.9033
6.8961
6.8879
6.8846
6.8808
6.8781
6.8691
6.8623
6.8468
6.8418
6.8307
6.8270
6.8068
3.8290
3.3032
3.2871
3.2711
1.9450
1.9365
1.9286
1.9206
1.9122
3.63
3.01
4.00
3.91
ppm

**4g**

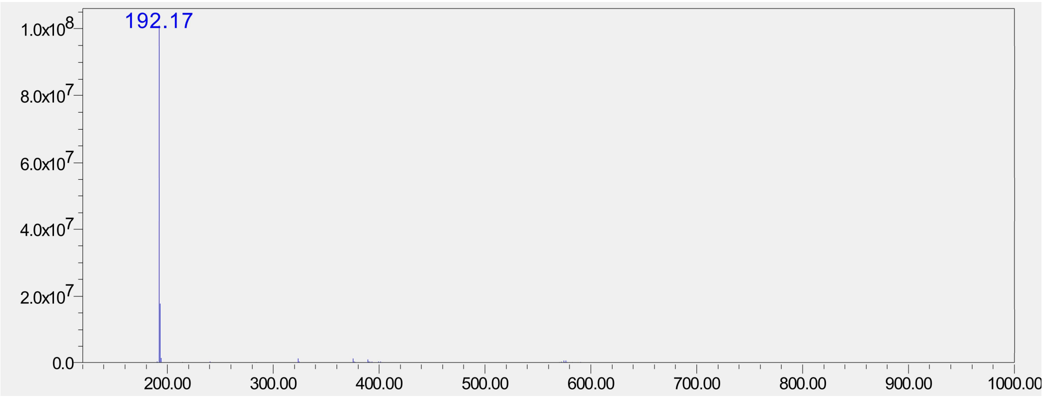
192.17

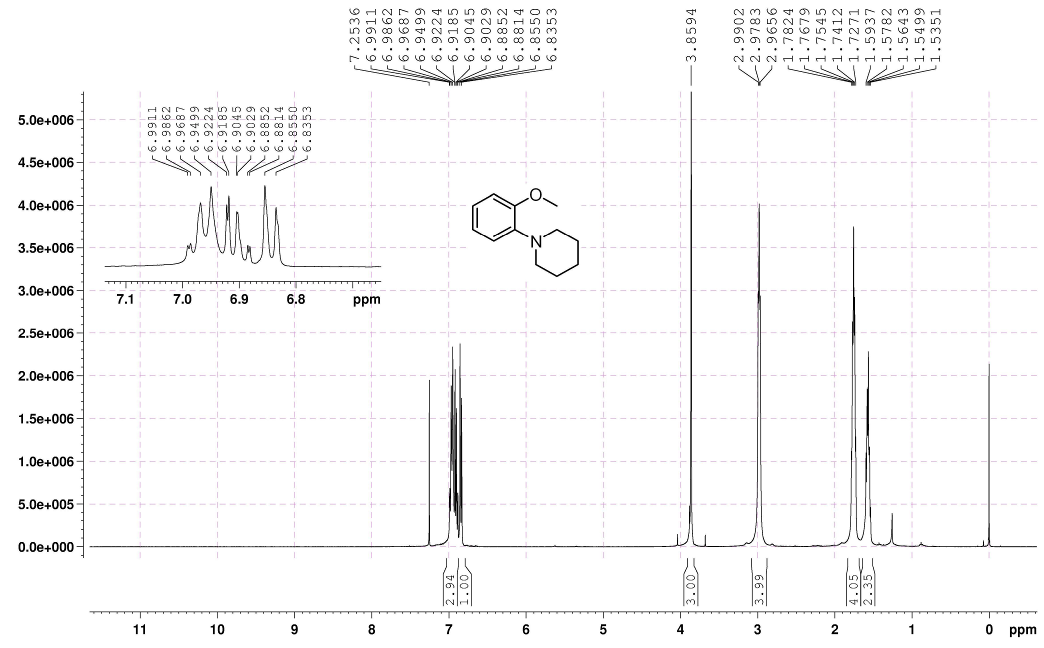
7.2536
6.9911
6.9862
6.9687
6.9499
6.9224
6.9185
6.9045
6.9029
6.8852
6.8814
6.8550
6.8353
3.8594
2.9902
2.9783
2.9656
1.7824
1.7679
1.7545
1.7412
1.7271
1.5937
1.5782
1.5643
1.5499
1.5351
2.94
1.00
3.00
3.99
4.05
2.35
ppm

**4h**

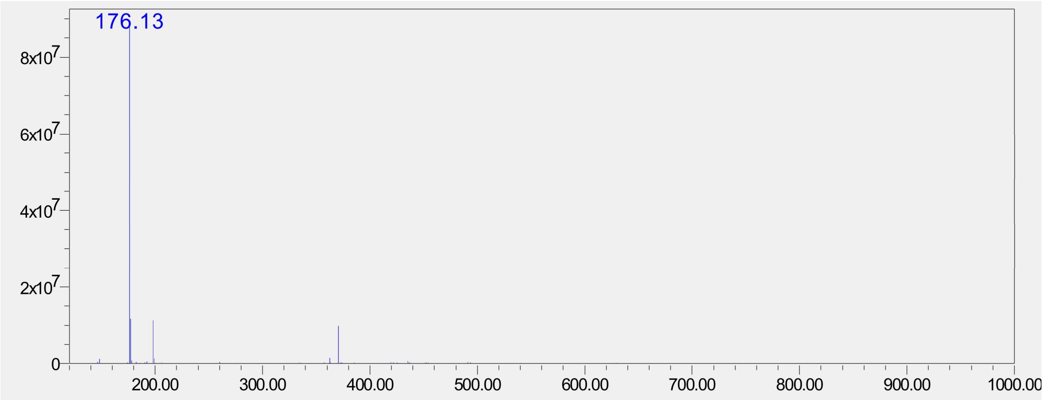
176.13

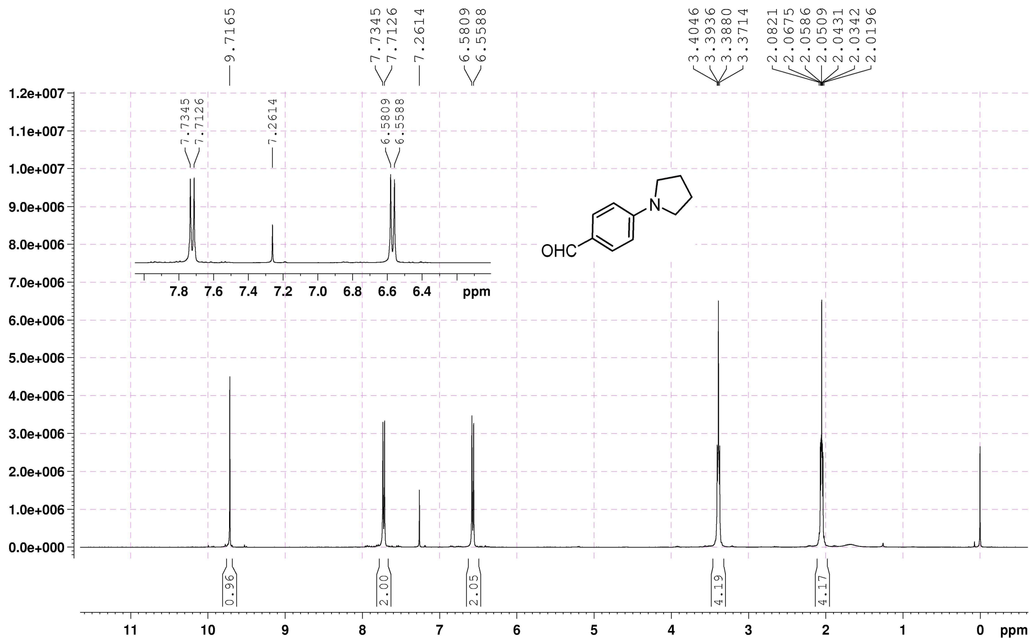

**4i**

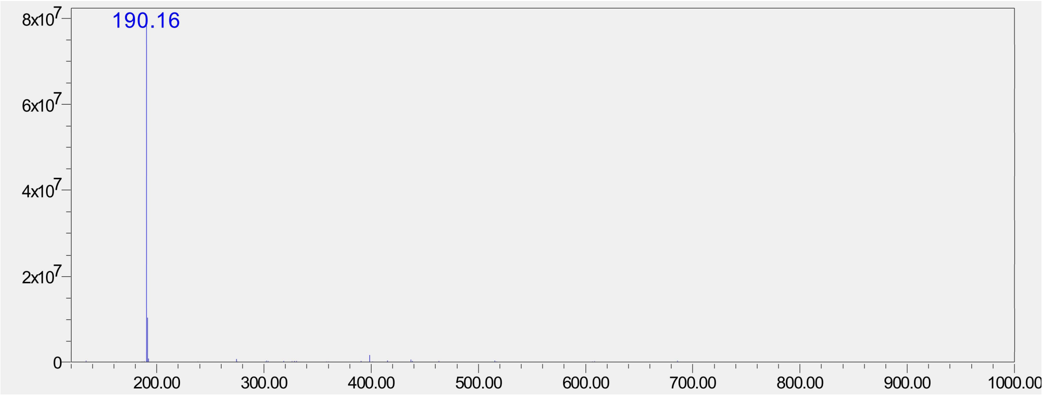

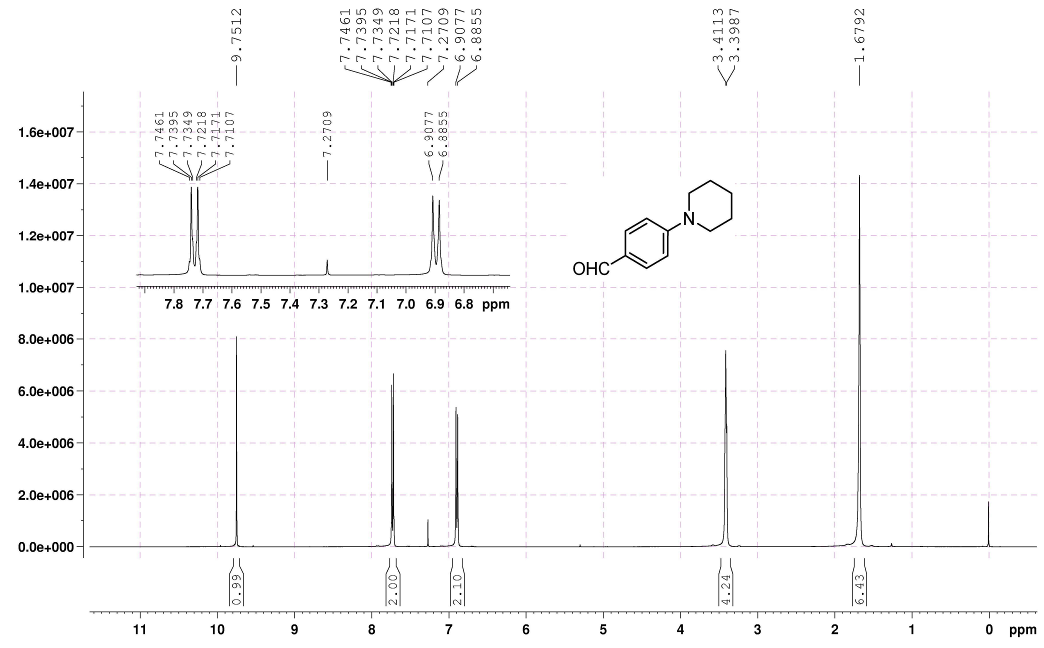

9.7512
7.7461
7.7395
7.7349
7.7218
7.7171
7.7107
7.2709
6.9077
6.8855
3.4113
3.3987
1.6792
OHC
N
ppm
0.99
2.00
2.10
4.24
6.43


**4j**

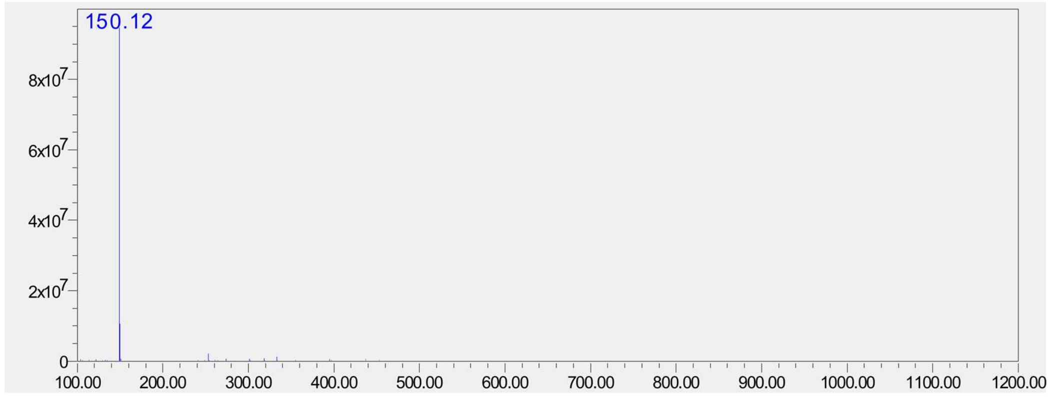

150.12

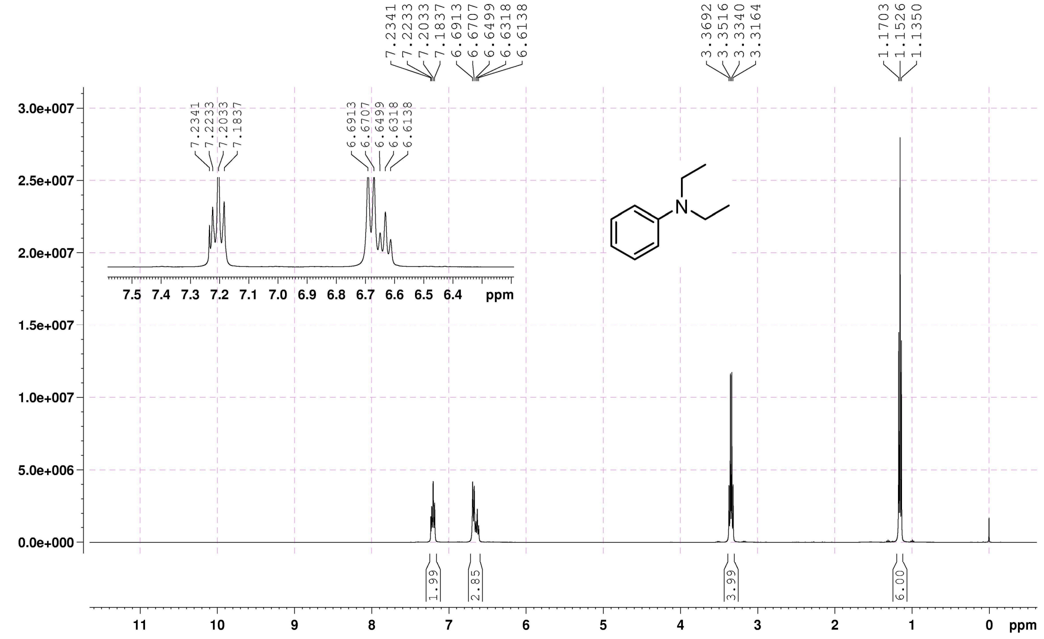
7.2341
7.2233
7.2033
7.1837
6.6913
6.6707
6.6499
6.6318
6.6138
3.3692
3.3516
3.3340
3.3164
1.1703
1.1526
1.1350
3.0e+007
2.5e+007
2.0e+007
1.5e+007
1.0e+007
5.0e+006
0.0e+000
7.5 7.4 7.3 7.2 7.1 7.0 6.9 6.8 6.7 6.6 6.5 6.4 ppm
N
1.99
2.85
3.99
6.00
11 10 9 8 7 6 5 4 3 2 1 0 ppm

**4k**

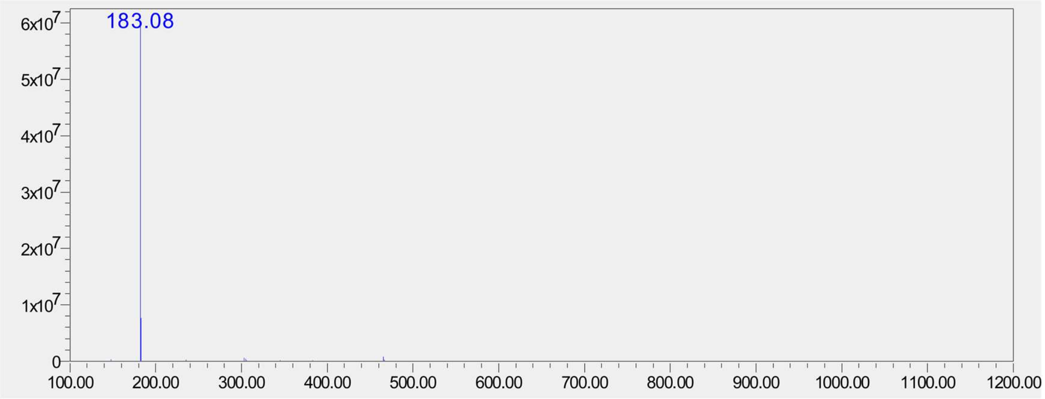
183.08
6x10^7
5x10^7
4x10^7
3x10^7
2x10^7
1x10^7
0
100.00 200.00 300.00 400.00 500.00 600.00 700.00 800.00 900.00 1000.00 1100.00 1200.00

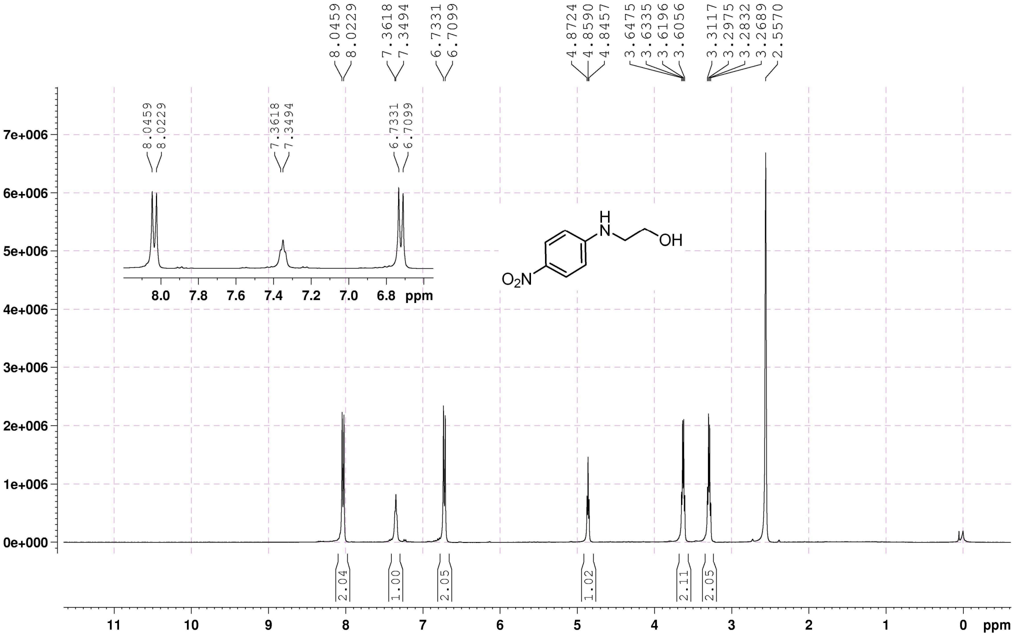
8.0459
8.0229
7.3618
7.3494
6.7331
6.7099
4.8724
4.8590
4.8457
3.6475
3.6335
3.6196
3.6056
3.3117
3.2975
3.2832
3.2689
2.5570
7e+006
6e+006
5e+006
4e+006
3e+006
2e+006
1e+006
0e+000
8.0
7.8
7.6
7.4
7.2
7.0
6.8
ppm
H
N
OH
$O_2N$
2.04
1.00
2.05
1.02
2.11
2.05
11
10
9
8
7
6
5
4
3
2
1
0
ppm

**4l**

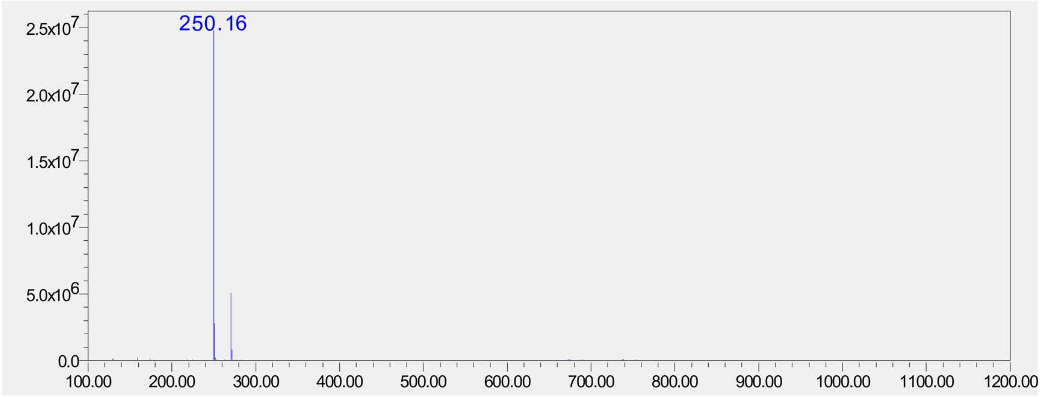
250.16
2.5x10^7
2.0x10^7
1.5x10^7
1.0x10^7
5.0x10^6
0.0
100.00
200.00
300.00
400.00
500.00
600.00
700.00
800.00
900.00
1000.00
1100.00
1200.00

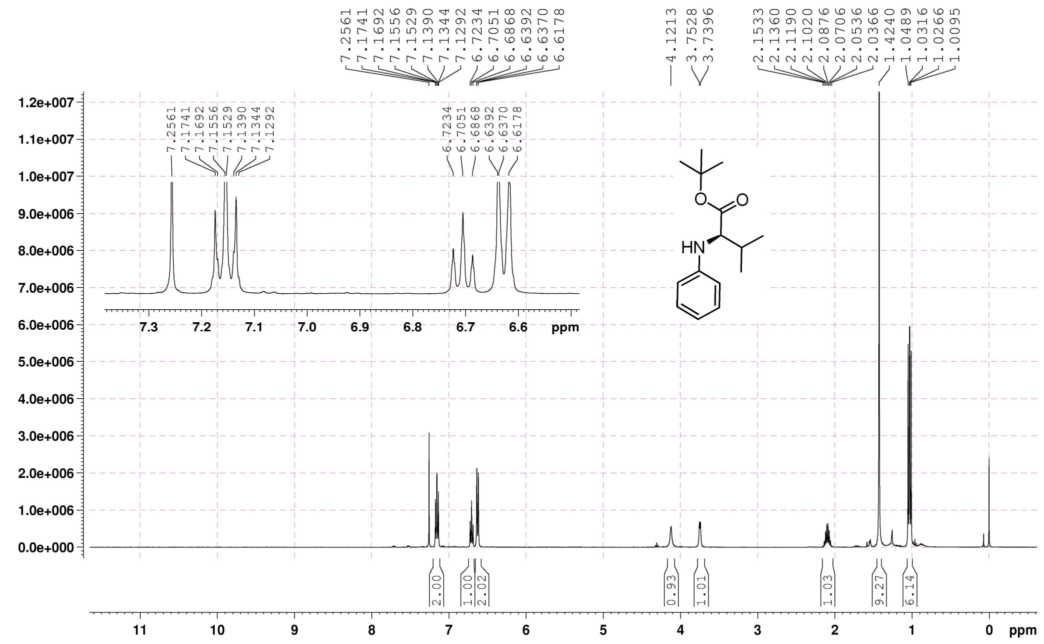
7.2561
7.1741
7.1692
7.1556
7.1529
7.1390
7.1344
7.1292
6.7234
6.7051
6.6868
6.6392
6.6370
6.6178
4.1213
3.7528
3.7396
2.1533
2.1360
2.1190
2.1020
2.0876
2.0706
2.0536
2.0366
1.4240
1.0489
1.0316
1.0266
1.0095
HN
O
2.00
1.00
2.02
0.93
1.01
1.03
9.27
6.14
ppm

**5a**

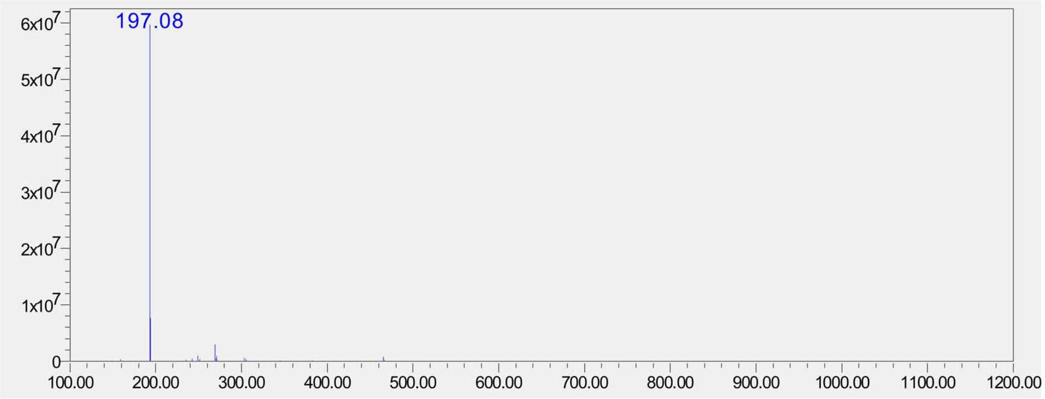
197.08

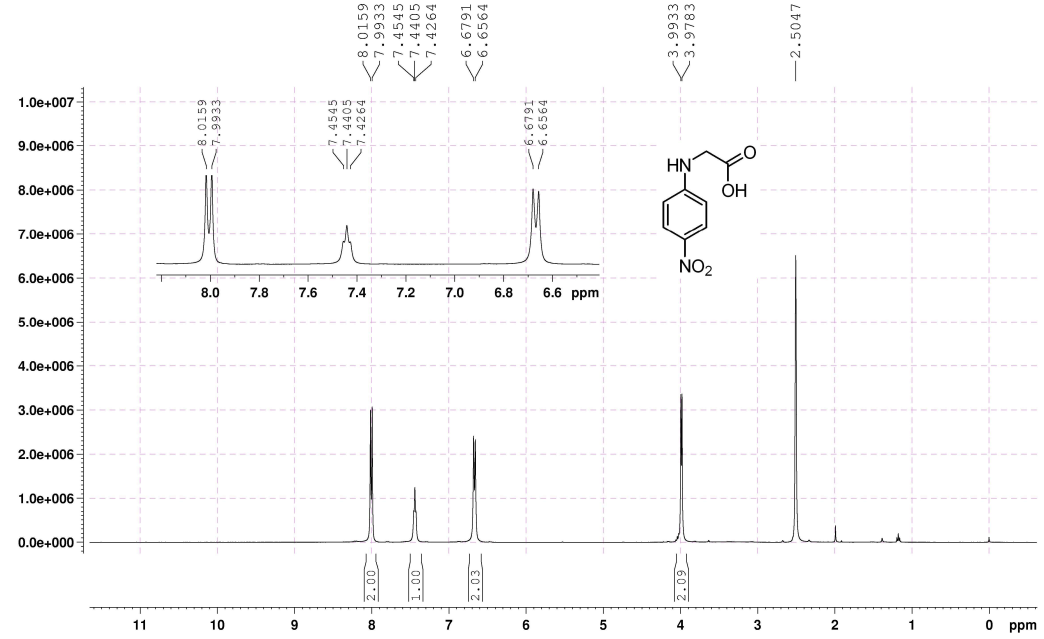

8.0159
7.9933
7.4545
7.4405
7.4264
6.6791
6.6564
3.9933
3.9783
2.5047
HN
O
OH
NO2
2.00
1.00
2.03
2.09
ppm


**5b**

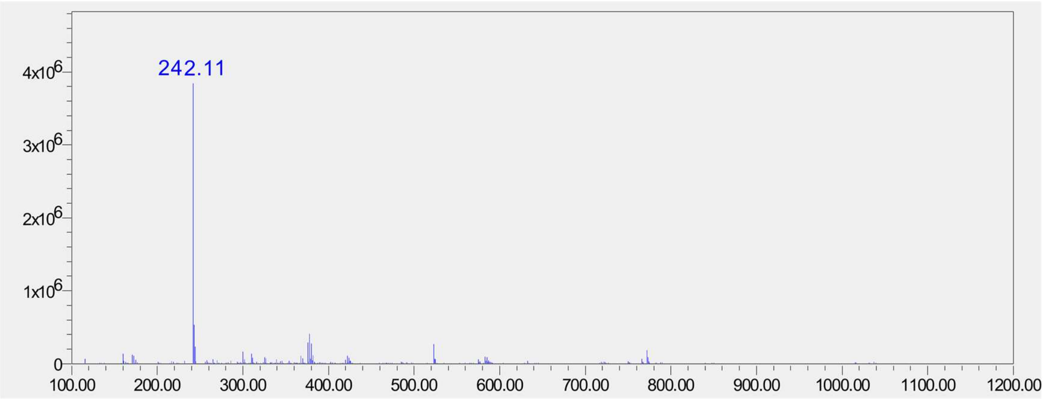

242.11
4x10^6
3x10^6
2x10^6
1x10^6
0
100.00
200.00
300.00
400.00
500.00
600.00
700.00
800.00
900.00
1000.00
1100.00
1200.00

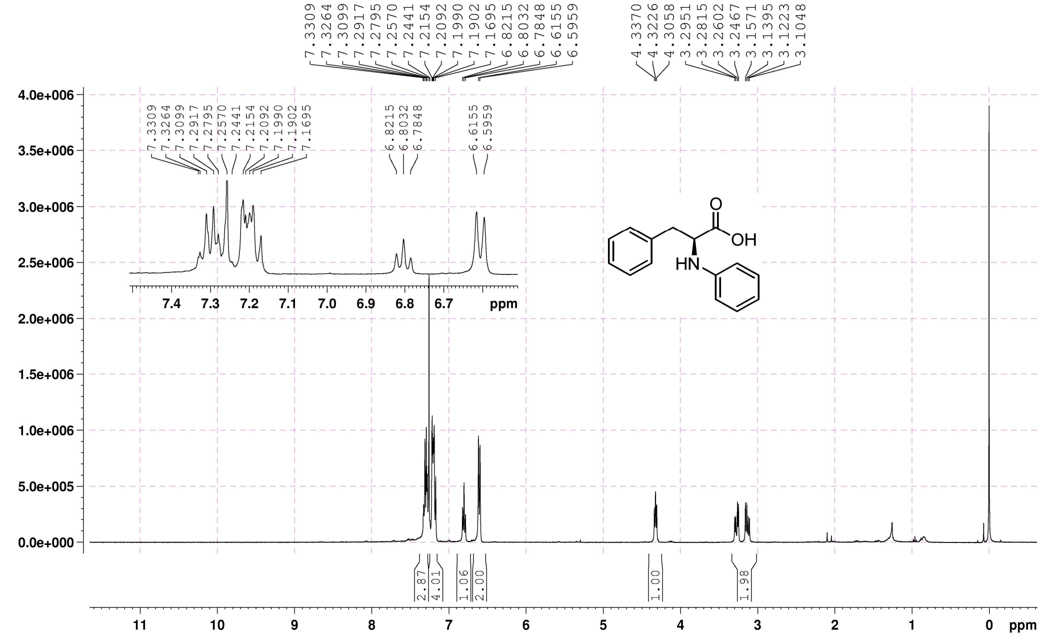

7.3309
7.3264
7.3099
7.2917
7.2795
7.2570
7.2441
7.2154
7.2092
7.1990
7.1902
7.1695
6.8215
6.8032
6.7848
6.6155
6.5959
4.3370
4.3226
4.3058
3.2951
3.2815
3.2602
3.2467
3.1571
3.1395
3.1223
3.1048
4.0e+006
3.5e+006
3.0e+006
2.5e+006
2.0e+006
1.5e+006
1.0e+006
5.0e+005
0.0e+000
OH
HN
2.87
4.01
1.06
2.00
1.00
1.98
ppm


**5c**

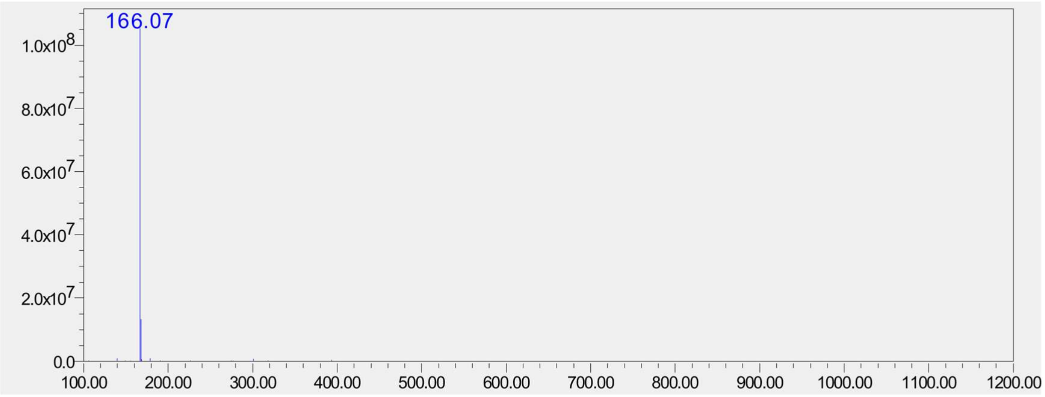

166.07
1.0x10^8
8.0x10^7
6.0x10^7
4.0x10^7
2.0x10^7
0.0
100.00
200.00
300.00
400.00
500.00
600.00
700.00
800.00
900.00
1000.00
1100.00
1200.00

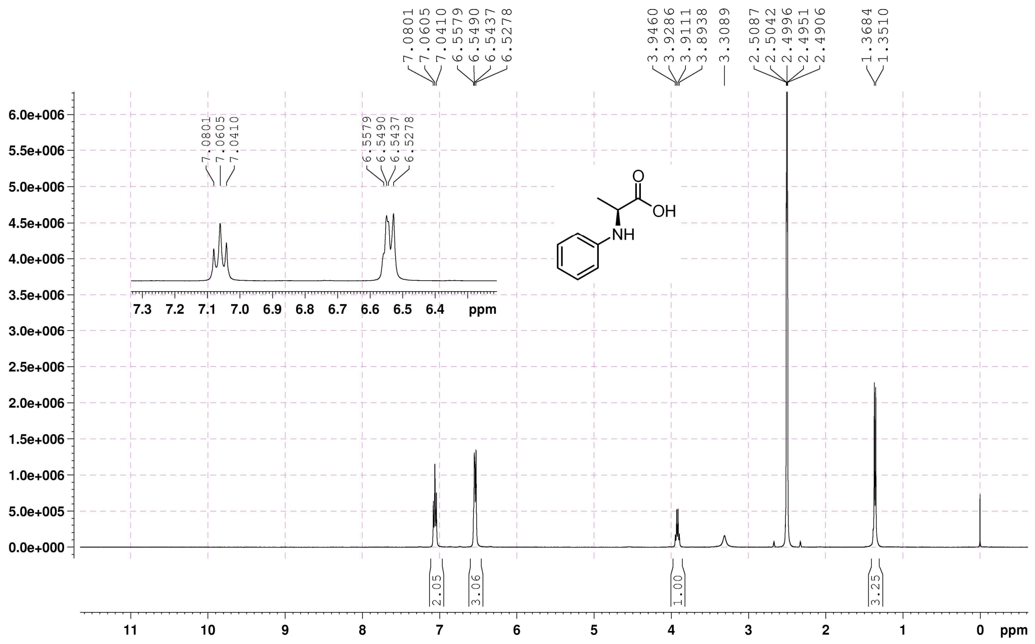
7.0801
7.0605
7.0410
6.5579
6.5490
6.5437
6.5278
3.9460
3.9286
3.9111
3.8938
3.3089
2.5087
2.5042
2.4996
2.4951
2.4906
1.3684
1.3510
O
OH
NH
2.05
3.06
1.00
3.25
ppm

**5d**

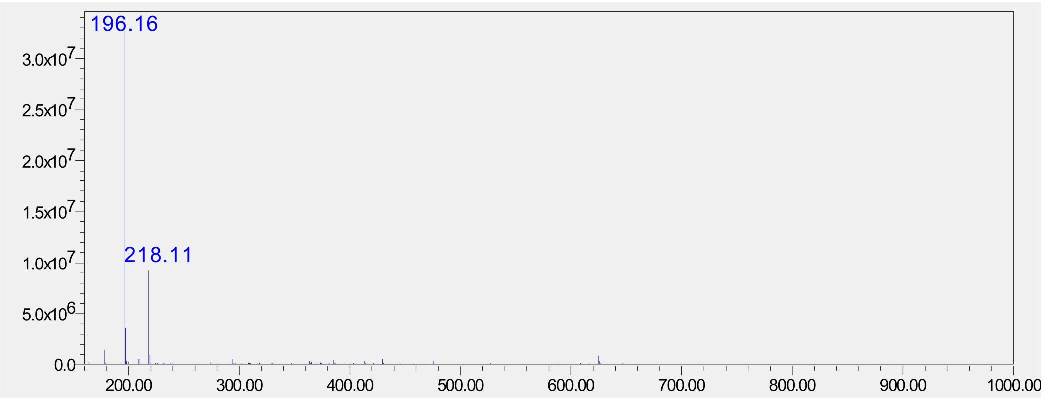
196.16
218.11

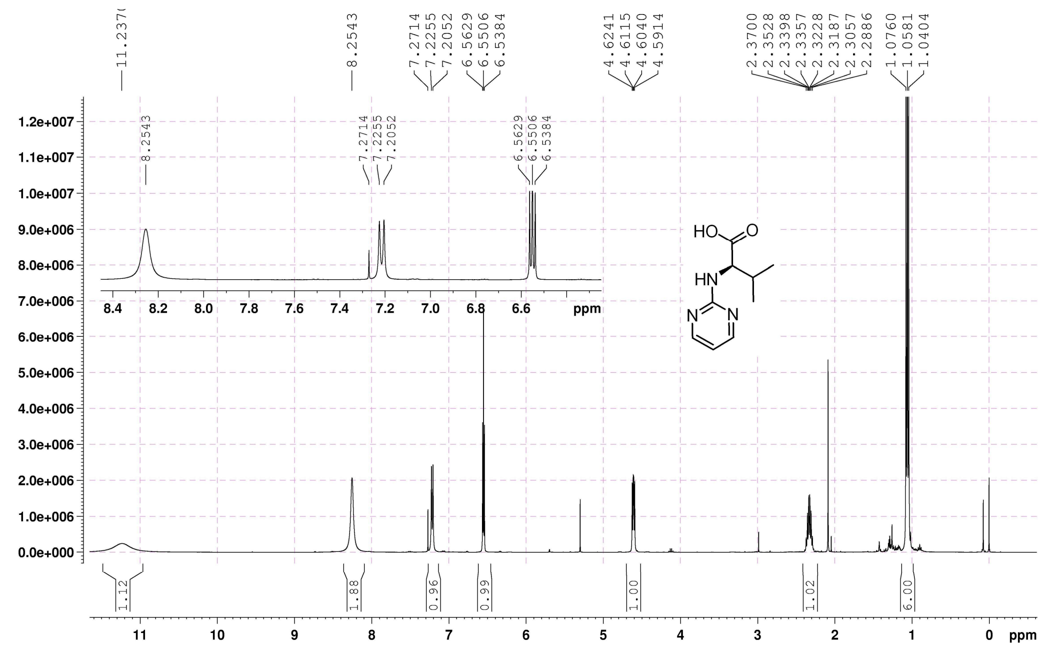

11.2370
8.2543
7.2714
7.2255
7.2052
6.5629
6.5506
6.5384
4.6241
4.6115
4.6040
4.5914
2.3700
2.3528
2.3398
2.3357
2.3228
2.3187
2.3057
2.2886
1.0760
1.0581
1.0404
HO
O
HN
N
N
1.12
1.88
0.96
0.99
1.00
1.02
6.00
ppm


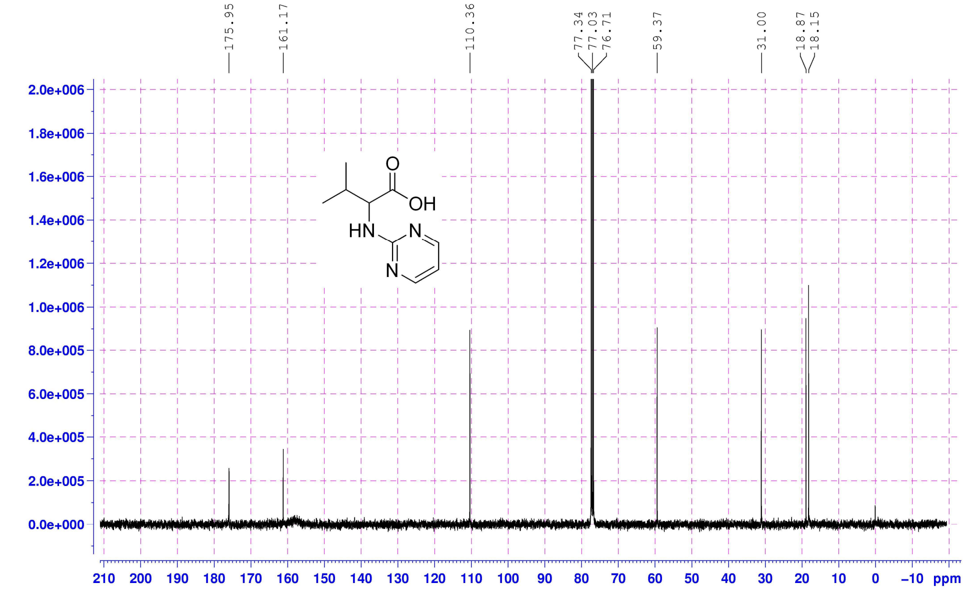

175.95
161.17
110.36
77.34
77.03
76.71
59.37
31.00
18.87
18.15
O
OH
HN
N
N
ppm

**5e**

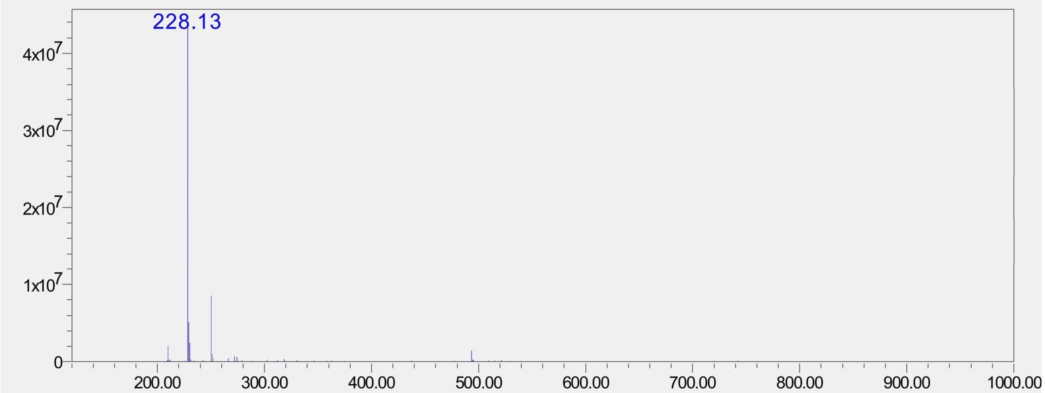
228.13
4x10^7
3x10^7
2x10^7
1x10^7
0
200.00
300.00
400.00
500.00
600.00
700.00
800.00
900.00
1000.00

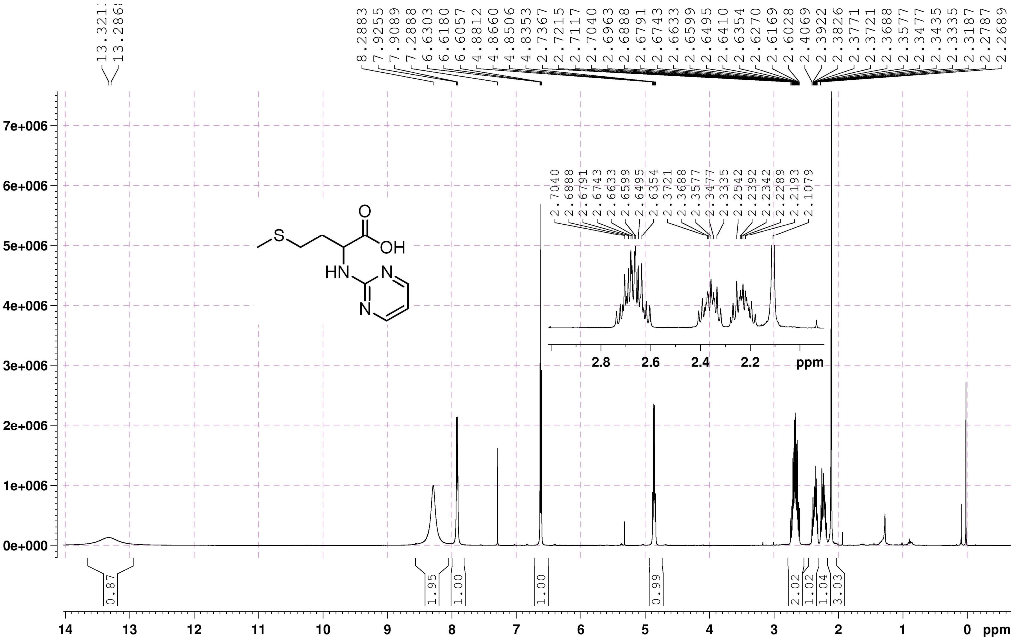
HN
N
N
S
O
OH
ppm

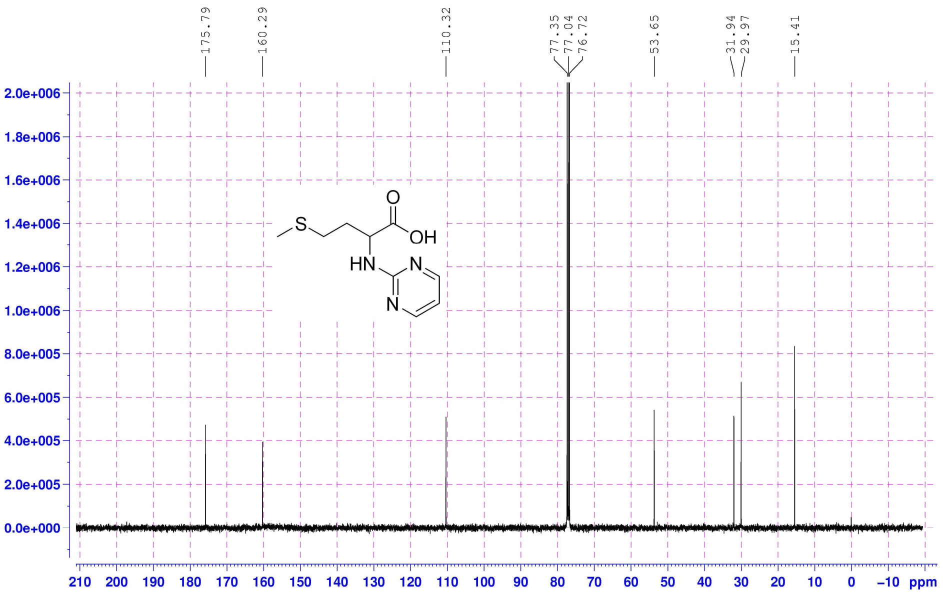
175.79
160.29
110.32
77.35
77.04
76.72
53.65
31.94
29.97
15.41
2.0e+006
1.8e+006
1.6e+006
1.4e+006
1.2e+006
1.0e+006
8.0e+005
6.0e+005
4.0e+005
2.0e+005
0.0e+000
210 200 190 180 170 160 150 140 130 120 110 100 90 80 70 60 50 40 30 20 10 0 −10 ppm
S
O
OH
HN
N
N

**5f**
